# How achievable are COVID-19 clinical trial recruitment targets? A UK observational cohort study and trials registry analysis

**DOI:** 10.1136/bmjopen-2020-044566

**Published:** 2020-10-05

**Authors:** Nick G Cunniffe, Simon J Gunter, Michael Brown, Sarah W Burge, Clare Coyle, Anthony De Soyza, Tom Dymond, Hanif Esmail, Darrel P Francis, Jacqui Galloway, James B Galloway, Effrossyni Gkrania-Klotsas, Jane Greenaway, George Katritsis, Prapa Kanagaratnam, Martin D Knolle, Kelly Leonard, Zoe C McIntyre, Ben Prudon, Tommy Rampling, Mili Estee Torok, Ben Warne, Mark Yates, Nicholas J Matheson, Li Su, Sofia Villar, Grant D Stewart, Mark Toshner

**Affiliations:** 1 Department of Clinical Neurosciences, Cambridge University, Cambridge, UK; 2 Harvard Medical School, Harvard University, Boston, Massachusetts, USA; 3 Division of Infection, University College London Hospital NHS Trust, London, UK; 4 Cancer Research UK Urological Malignancies Programme, Department of Oncology, University of Cambridge, Cambridge, UK; 5 Department of Cardiology, Hammersmith Hospitals NHS Trust, London, UK; 6 Respiratory Medicine, Freeman Hospital, Newcastle upon Tyne, UK; 7 Department of Infection and Inflammation Research, Cambridge University Hospitals NHS Foundation Trust, Cambridge, UK; 8 Hospital for Tropical Diseases, University College London Hospitals NHS Foundation Trust, London, UK; 9 MRC Clinical Trials Unit, University College London, London, UK; 10 Institute for Global Health, University College London, London, United Kingdom; 11 Faculty of Medicine, National Heart & Lung Institute, Imperial College London, London, UK; 12 Department of Respiratory Medicine, Cambridge University Hospitals NHS Foundation Trust, Cambridge, UK; 13 Centre for Rheumatic Diseases, Kings College London, London, UK; 14 Department of Infectious Diseases, Cambridge University Hospitals NHS Foundation Trust, Cambridge, UK; 15 Research and Development, North Tees Hospital, Stockton-on-Tees, UK; 16 National Heart and Lung Institute, Imperial College London, London, UK; 17 Department of Cardiology, St Marys Hospital, Imperial College Healthcare NHS Trust, London, UK; 18 Cambridge Urology Translational Research and Clinical Trials Department, Cambridge University Hospitals NHS Foundation Trust, Cambridge, UK; 19 School of Clinical Medicine, Office for Translational Research, University of Cambridge, Cambridge, UK; 20 Department of Respiratory Medicine, North Tees Hospital, Stockton-on-Tees, UK; 21 Division of Pathology, University College London Hospital NHS Trust, London, United Kingdom; 22 University of Cambridge, Cambridge, UK; 23 Cambridge Institute of Therapeutic Immunology and Infectious Disease, University of Cambridge, Cambridge, UK; 24 NHS Blood and Transplant, Cambridge, UK; 25 Department of Medicine, University of Cambridge, Cambridge, UK; 26 MRC Biostatistics Unit, University of Cambridge School of Clinical Medicine, Cambridge Institute of Public Health, Cambridge, United Kingdom; 27 Department of Surgery, University of Cambridge, Cambridge, UK; 28 NIHR Respiratory Translational Research Collaboration, Cambridge, UK

**Keywords:** COVID-19, clinical trials, infectious diseases

## Abstract

**Objectives:**

To analyse enrolment to interventional trials during the first wave of the COVID-19 pandemic in England and describe the barriers to successful recruitment in the circumstance of a further wave or future pandemics.

**Design:**

We analysed registered interventional COVID-19 trial data and concurrently did a prospective observational study of hospitalised patients with COVID-19 who were being assessed for eligibility to one of the RECOVERY, C19-ACS or SIMPLE trials.

**Setting:**

Interventional COVID-19 trial data were analysed from the clinicaltrials.gov and International Standard Randomized Controlled Trial Number databases on 12 July 2020. The patient cohort was taken from five centres in a respiratory National Institute for Health Research network. Population and modelling data were taken from published reports from the UK government and Medical Research Council Biostatistics Unit.

**Participants:**

2082 consecutive admitted patients with laboratory-confirmed SARS-CoV-2 infection from 27 March 2020 were included.

**Main outcome measures:**

Proportions enrolled, and reasons for exclusion from the aforementioned trials. Comparisons of trial recruitment targets with estimated feasible recruitment numbers.

**Results:**

Analysis of trial registration data for COVID-19 treatment studies enrolling in England showed that by 12 July 2020, 29 142 participants were needed. In the observational study, 430 (20.7%) proceeded to randomisation. 82 (3.9%) declined participation, 699 (33.6%) were excluded on clinical grounds, 363 (17.4%) were medically fit for discharge and 153 (7.3%) were receiving palliative care. With 111 037 people hospitalised with COVID-19 in England by 12 July 2020, we determine that 22 985 people were potentially suitable for trial enrolment. We estimate a UK hospitalisation rate of 2.38%, and that another 1.25 million infections would be required to meet recruitment targets of ongoing trials.

**Conclusions:**

Feasible recruitment rates, study design and proliferation of trials can limit the number, and size, that will successfully complete recruitment. We consider that fewer, more appropriately designed trials, prioritising cooperation between centres would maximise productivity in a further wave.

**Strengths and limitations of this study**

- We comprehensively analysed clinical trial registry data to quantify the number of participants required to successfully complete enrolment to interventional COVID-19 trials based in England in the first wave of the pandemic.
- We simultaneously performed a large, prospective, observational cohort study of 2082 people hospitalised with COVID-19 to report recruitment rates across a range of secondary and tertiary centres and characterise reasons for trial exclusion.
- Using government data on COVID-19 hospitalisations, we consider the differences between the trials community’s aspirations and delivery, and how this might inform our strategy in the event of a second wave.
- Our analysis is restricted to two registry databases and includes trials that started recruiting late in the first wave; we therefore likely underestimate the recruitment target and overestimate the number of eligible patients.
- Our analysis is limited to data based in England and, while we consider global trials, our conclusions may not be representative of, or readily translatable to, international cohorts.

## Introduction

Unless a successful vaccination programme is deployed, the greatest need for COVID-19 remains effective treatments. This presents a substantial challenge. Ostensibly, the response from the experimental medicine community to the first wave has been robust, with >1970 clinical trials planned, recruiting or completed, at the time of writing.^1^ This has enabled enrolment of patients to trials of drugs with known safety profiles—including lopinavir,^2^ remdesivir,^3 4^ hydroxychloroquine^5 6^ and tocilizumab^7^—and led to positive results, such as the 12.1% absolute risk reduction in mortality among ventilated patients treated with dexamethasone.^8^

However, while many of these trials have been pragmatic in terms of selection criteria, the proportion of hospitalised patients with COVID-19 being recruited to clinical trials is lower than might have been anticipated; the authors of the RECOVERY trial recently estimated a 10% recruitment rate in the UK.^9^ Meanwhile, in areas where public health measures have limited viral transmission, trials have terminated early on account of under-recruitment.^10 11^ With mounting concern about an ensuing second wave of infection,^12 13^ it is increasingly important to learn lessons from the first, and consider the number, size and design of clinical trials that can feasibly be completed.

We hypothesised that the proliferation of SARS-CoV-2 interventional studies during the pandemic and under recognised barriers to recruitment of patients with COVID-19 led to unachievable recruitment targets in England. We used data from clinical trial registry databases to quantify recruitment targets and concurrently studied recruitment rates, including reasons for exclusion, across five centres enrolling patients at the peak of the first wave of the pandemic. In conjunction with publicly available data from the UK government, we consider the differences between the trials community’s aspirations and delivery, and how this might inform our strategy if there were a second wave.

## Methods

### Establishing recruitment targets for registered trials during first wave

COVID-19 clinical studies registered on clinicaltrials.gov or the International Standard Randomized Controlled Trial Number (ISRCTN) databases were identified and study data downloaded on 12 July 2020. Data for trials based in England, multinational trials with centres in England and global trials were extracted in turn. Cross-registered studies were identified and accounted for once in the analysis. A manual review determined whether sponsors were academic, non-academic or mixed. Trials were excluded if they were labelled as terminated, withdrawn or suspended. Data for interventional trials examining treatment and prevention were documented, but only trials of COVID-19 treatments were used in the analysis. Analyses were performed using RStudio V.1.2.5042.

### Observational study of recruitment of hospitalised patients

We performed a prospective observational study of 2082 consecutive patients with SARS-CoV-2 infection at five hospitals affiliated to the National Institute for Health Research-Translational Research Collaboration with representation from secondary and tertiary centres: Cambridge University Hospitals NHS Foundation Trust (CUHFT), Cambridge; Imperial College Healthcare, University College Hospital and King’s College Hospitals, London and University Hospital of North Tees, Middlesbrough. Subjects were admitted and eligibility assessed for: RECOVERY (ISRCTN50189673), C19-ACS (NCT04333407) or SIMPLE (NCT04292730/NCT04292899). CUHFT local R&D approval was undertaken.

Demographic and clinical data were collected by contemporaneous review of potential participants’ case notes. A categorical approach subdivided primary reasons subjects were not enrolled into: (a) clinical grounds (screening or treating physician judgement that comorbidity or other reason for admission was more critical to patient outcome than COVID-19), (b) medically fit for discharge, (c) receiving end-of-life care, (d) lack of capacity, (e) patient refusal, (f) interactions with trial drugs or (g) already on mechanical ventilation. Although already being on mechanical intervention was not an exclusion criterion for RECOVERY, patients categorised as excluded on these grounds were ineligible on account of competing, intensive care-based, studies.

### Establishing feasible recruitment for registered trials during first wave

Using publicly available UK government data of the numbers of patients with COVID-19 admitted to English hospitals during the first wave between 17 March and 5 August 2020,^14^ and the recruitment rate (with 95% CI for one sample proportion with continuity correction) from the aforementioned observational study, we estimated a maximum bound for the accumulated feasible recruitment during that time. Simultaneously, we used the estimated cumulative number of infected cases in England by 12 July provided by Medical Research Council (MRC) Biostatistics Unit at the University of Cambridge^15^ to calculate an approximate hospitalisation rate in England among COVID-19 infections. We based our estimates on data from centres in England as the infection rate estimates were more reliable, hospitalisation criteria were different in Wales^14^ and the five hospitals included in this study are all from England.

### Patient and public involvement

This was a time-critical study in response to a Public Health Emergency of International Concern. Patients or the public were not involved in the design, conduct or reporting of this research.

## Results

### Establishing recruitment targets to registered trials during first wave

Clinical trial registry data were downloaded on 12 July 2020; 28 interventional studies were included in our analysis of those registered in England. Twenty-two (78%) were academically sponsored, 5 (18%) were non-academically sponsored and 1 (4%) was mixed. The first registration date of a COVID-19 treatment trial in England was 22 March; the earliest registered start date was 12 March. Analysis of recruitment targets for each trial revealed that 46 154 participants would be required to complete recruitment to all studies in England (table 1): 17 012 people are required for trials of prophylactic drugs to prevent COVID-19, while 29 142 are needed for those treating established COVID-19 (table 1). The median (IQR) treatment trial recruitment target was 195 (50–793).

**Table 1:** Summary of number of trials and required numbers of participants

	Number of trials	Number of participants
Global trials		
Prevention	172	260 446
Treatment	935	306 426
Total	1107	566 872
UK multinational and national trials		
Prevention	11	97 272
Treatment	38	44 362
Total	49	141 634
England trials		
Prevention	8	17 012
Treatment	20	29 142
Total	28	46 154

**Figure 1:**
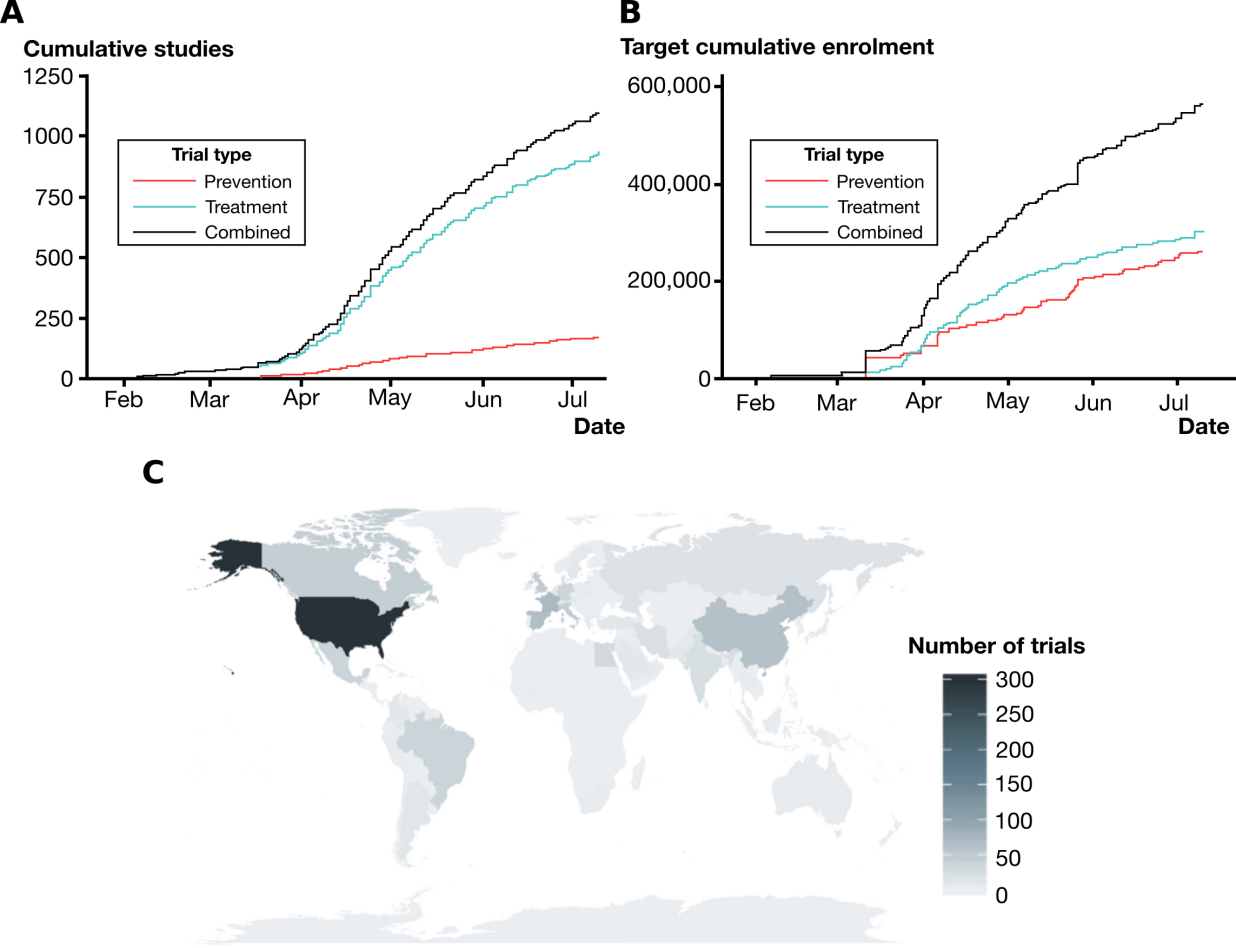
The proliferation of global clinical trials in response to COVID-19. (A) Cumulative number of enrolling studies registered with clinicaltrials.gov or International Standard Randomized Controlled Trial Number until 12 July 2020, subdivided by those testing drugs for COVID-19 treatment and prevention. (B) Cumulative number of participants required to meet recruitment targets for registered clinical trials. (C) Geographical distribution of COVID-19 clinical trials.

By contrast, the global situation is such that 1107 registered interventional trials were ongoing or completed, requiring 566 872 patients to be randomised to allow their completion; 306 426 of these are needed for trials of COVID-19 treatments (figure 1A, B). These trials are geographically clustered in China, North America and Europe (figure 1C).

### Observational study of clinical trial enrolment

From 27 March to 22 May 2020, a total of 2082 consecutive patients were included across the five sites (table 2). Age and sex data were available for 1971 patients: the median (IQR) age was 71 (58–82) and 56.2% were male. Across the four trials, 430 (20.7%, 95% CI 18.95% to 22.47%) proceeded to randomisation.

**Table 2:** Screening data for 2082 consecutive patients with laboratory-confirmed SARS-CoV-2 admitted to one of five centres

	RECOVERY				Combination*	C19-ACS	SIMPLE	Total
**Total screened per centre**	281	83	415	Total (779)	445	784	74	2082
**Number recruited (%)**	35 (12.5)	16 (19.3)	185 (44.6)	236 (30.3)	124 (27.9)	56 (7.1)	14 (18.9)	**430 (20.7)**
Refused participation (%)	10 (3.6)	19 (22.9)	16 (3.9)	45 (5.8)	8 (1.8)	29 (3.7)	0 (0.0)	82 (3.9)
Clinical grounds/trial exclusion criteria(%)	83 (29.5)	15 (18.1)	40 (9.6)	138 (17.7)	167 (37.5)	365 (46.6)	29 (39.2)	699 (33.6)
Lacked capacity (%)	22 (7.8)	0 (0.0)	1 (0.2)	23 (3.0)	16 (3.6)	98 (12.5)	0 (0.0)	137 (6.6)
Mechanical ventilation (%)	37 (13.2)	7 (8.4)	0 (0.0)	44 (5.6)	7 (1.6)	48 (6.1)	7 (9.5)	106 (5.1)
Drug interactions (%)	12 (4.3)	2 (2.4)	0 (0.0)	14 (1.8)	2 (0.4)	1 (0.1)	0 (0.0)	17 (0.8)
Medically fit for discharge (%)	55 (19.6)	14 (16.9)	77 (18.6)	146 (18.7)	65 (14.6)	136 (17.3)	16 (21.6)	363 (17.4)
Palliative care (%)	19 (6.8)	7 (8.4)	61 (14.7)	87 (11.2)	8 (1.8)	51 (6.5)	7 (9.5)	153 (7.3)
Not approached or considered (%)	8 (2.8)	3 (3.6)	35 (8.4)	46 (5.9)	48 (10.8)	0 (0.0)	1 (1.4)	95 (4.6)
**Total not recruited (%)**	246 (87.5)	67 (80.7)	230 (55.4)	543 (69.7)	321 (72.1)	728 (92.9)	60 (81.1)	**1652 (79.3)**

*Centre screened concurrently to both RECOVERY and SIMPLE: moderate and severe trials.

Of the remaining 1652 patients, 82 (3.9%) declined participation, 363 (17.4%) were medically fit for discharge, 153 (7.3%) were receiving end-of-life care and 106 (5.1%) were mechanically ventilated at the time of screening. In 699 (33.6%) patients, the screening or treating physician determined that the potential participant should not be enrolled on account of clinical grounds or trial exclusion criteria.

### Establishing feasible recruitment for registered trials during first wave

**Figure 2:**
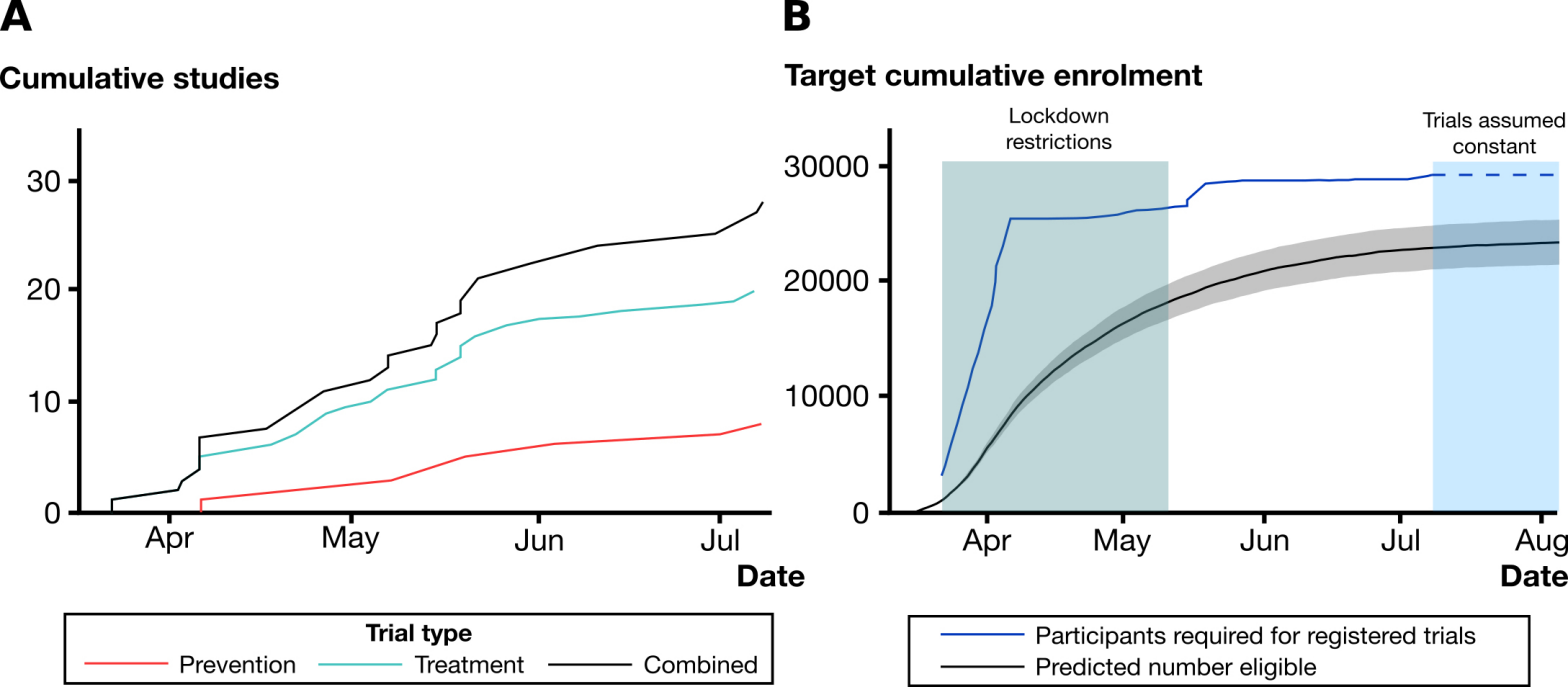
Feasibility of achieving target recruitment in England for COVID-19 interventional studies. (A) Cumulative number of enrolling studies in England registered with clinicaltrials.gov or International Standard Randomized Controlled Trial Number until 12 July 2020, subdivided by those testing drugs for COVID-19 treatment and prevention. (B) Cumulative number of participants required to meet recruitment targets for registered COVID-19 treatment trials until 12 July 2020, and predicted number of patients who would have been eligible for randomisation (grey shaded area represents point-wise 95% confidence band for the predictive cumulative number of eligible patients using the lower and upper value of 95% CI for the recruitment rate estimate with continuity correction). The reduction in the infection rate in England means that the recruitment target at 12 July is unlikely to be reached unless there is a second wave; further illustrated by extending hospitalisation data to 5 August 2020.

By combining these observed recruitment rates with publicly reported hospitalisation data (between 17 March and 12 July 2020), we estimated a maximum upper bound for the accumulated feasible recruitment for registered trials of COVID-19 treatments in England during the first wave (figure 2A, B).

The estimated number of cumulative infected cases by 12 July reported by MRC Biostatistics Unit is 4.67 million with a 95% credible interval (3.76 to 6.04). Combined with the number of cumulative admitted patients in England by 12 July from government data (ie, 111 037 hospital admissions), this gives an approximate hospitalisation rate 2.38% (1.84% to 2.95%) in England during the first wave.

Our analysis indicates that by 12 July, 6158 patients might still be needed to meet the total recruitment targets for currently recruiting clinical trials. If considering uncertainty in recruitment rate estimate reflected by 95% CI (18.95% to 22.47%), 4192–8100 patients might be required to meet recruitment target. Assuming the recruitment rate 20.7%, this implies that 29 749 hospitalised patients would need to be screened for these trials to complete recruitment. With the approximate hospitalisation rate 2.38% in England as observed in the first wave, this would require 1.249 million patients to be infected.

With the daily infection rate for UK estimated to be 3310 (95% credible interval (2440 to 4460)) on 12 July,^15^ it is highly unlikely such a large number of hospitalisations would occur unless there is an increase in the infection numbers (or a second wave). Indeed, incorporating hospitalisation data to 5 August 2020 shows minimal progress towards the recruitment target, assuming no new trials were approved after 12 July 2020 (figure 2A, B).

## Discussion

We found that the proliferation of clinical trials^1^ in response to the first wave of the COVID-19 pandemic in England required 29 142 participants to complete enrolment to those registered with a trials database. Globally, 306 426 participants are required to meet recruitment targets for trials of treatments of COVID-19. Meanwhile, in our multicentre prospective observational cohort study of patients admitted to hospital with laboratory-confirmed COVID-19, 79.3% of potential participants were not recruited to a clinical trial; the reasons for excluding patients were varied and clarify the challenges faced in both general hospitals and well-resourced centres experienced in experimental medicine. Our experience is consistent with the general literature on clinical trial recruitment where many factors have been posited to contribute to heterogeneity of recruitment.^16^ With 111 037 people hospitalised in England between 17 March and 12 July 2020, our net recruitment rate suggests that 22 985 (21 042–24 950 if taking into account uncertainty in recruitment rate estimate by random errors) would have been potentially suitable for selection in the first wave. However, this is clearly an overestimate, given that it would require each of these individuals to be hospitalised in geographical locations where medical centres were undertaking these trials. In the first wave, most general clinical trials infrastructure was mothballed for normal activity and therefore easily seconded towards COVID-19 and this may not be the case in subsequent ‘waves’. It must also be recalled that most recruitment in the first wave was undertaken as hospitals were actively reconfiguring services. A stable hospital infrastructure may positively impact on ease of delivery in the future. Nevertheless, unless there is a second wave it is highly unlikely that the total recruitment target will be met in any reasonable timeframe.

Strengths of our study are that our analyses of registry and population databases used the largest and most robust data available. Meanwhile, our observational study applied a large cohort size, prospective data acquisition and recorded detailed reasons for excluding patients. By using both secondary and tertiary care centres, we believe our results are generalisable to other hospitals in the UK. Also, by following studies with minimal selection criteria, particularly in the RECOVERY trial, we reduced the chance of underestimating trial recruitment. Our study does have limitations. First, our predictions were based on registry data for studies based in England alone; we did not include the numbers of participants required to be recruited into multinational trials in which the English centres were involved. The result is that we have likely underestimated the trial recruitment target for England and, by extension, the gap between this and the number of participants available. Second, although we used hospitalisation data from 17 March 2020, as this was the time the UK government commenced public reporting of COVID-19 admissions, all trials included in our registry analysis were not recruiting at that stage; the earliest start date for a trial registered in England was 12 March 2020, but the last trial start date was not until 7 July 2020. In this sense, using cumulative number of admitted patients in our prediction is optimistic. Third, we only included the two registry datasets in most widespread use, and so may have further underestimated the number of studies and participants required. Fourth, the 95% CI for recruitment rate estimate only reflects the uncertainty due to random errors in the data, it does not consider the uncertainty due to unrepresentativeness of data from the five hospital centres in our study. Finally, although we illustrate the scale of trial recruitment required globally, the populations tested may not be representative of, or translatable to, international cohorts.

Our study is the first to characterise the suitability and barriers for trial enrolment for a complete cohort of hospitalised patients with COVID-19. Results of trials published to date convey a different message: interventional studies of lopinavir and remdesivir, for example, have recruitment rates ranging from 55.7% to 96.0%.^2–4^ This difference is most likely explained by the different denominators used in our calculations: the consort diagrams in clinical trials are unlikely to include every single patient hospitalised with a positive test. Instead, our results align with or exceed other centres, such as the 10% recruitment rate to RECOVERY.^9^ During the 2013–16 Ebola Virus Disease epidemic in west Africa, most clinical trials during that crisis either started too late to enrol sufficient case numbers or were simply unable to reach their recruitment targets.^17^ Our study shows that trials in England started recruiting relatively quickly, however many are highly unlikely to recruit on time; we conclude that starting early is important but not enough to ensure recruitment targets are met. Finally, it is notable that our calculated hospitalisation rate of 2.38% is lower than that observed in Wuhan,^18^ which if applied to the UK age structure,^19^ is equivalent to approximately 5.8%.

The disparity between the realistic recruitment rates and high requirements we report leads us to conclude that the scientific community should be increasingly selective in the number, size and design of clinical trials deployed in the COVID-19 pandemic; our findings have meaning for those planning single trials, and those strategising the national response. Potential solutions include practical changes to trial design, for instance, capturing patients earlier in their disease path, and adopting dynamic and adaptive trial designs.^20^ Yet, such measures are unlikely to bridge the currently estimated large recruitment gap. Instead, it may be necessary for healthcare authorities and policy makers to foster more academic cooperation to prioritise compounds, prevent duplication and, perhaps more radically, perform real-time meta-analyses of ongoing trials of the same therapies and provide stop/go recommendations across trials to rationalise treatment and prevent multiple studies delaying reporting.^21^ Indeed, proposals have been forthcoming for mechanisms by which data from different trials might be shared and analysed in a robust and scientifically meaningful way.^22^ These conclusions are not dissimilar to reflections from the Ebola pandemic, when there was a strong call for strengthening and coordinating research efforts in response to outbreaks of emerging infectious diseases.^23 24^ For planning future trials and deriving realistic recruitment targets, real-time tracking of the pandemic, as data accumulate over time, is essential to plan research in response of an emerging epidemics outbreak. The MRC Biostatistics Unit regularly nowcast and forecast COVID-19 infections and deaths.^15^ This information feeds directly to SAGE subgroup, Scientific Pandemic Influenza subgroup on Modelling and to regional Public Health England teams. These same data could be used to establish realistic recruitment trends to inform, monitor and coordinate research efforts both for treatment and prevention trials.

Multiple questions remain for future research. In particular, it remains unclear how relaxing of non-pharmacological interventions will affect transmission rates, and therefore the achievability of remaining recruitment to these trials. It is also unknown how a second wave would evolve, and whether more or fewer people will develop the illness than was seen in the first. Nonetheless, we conclude that clinical trialists and healthcare authorities must consider the recruitment challenges when determining the feasibility of clinical trials in a second wave and urgently rationalise those currently active.

## Supplementary Material

Reviewer comments

Author's manuscript
