# Supplementary material for: How achievable are COVID-19 clinical trial recruitment targets? A UK observational cohort study and trials registry analysis
Source: BMJ Open. 2020 Oct 5;10(10):e044566. doi: 10.1136/bmjopen-2020-044566 (PMC7536634; doi:10.1136/bmjopen-2020-044566)
Supplement: Author's manuscript [file bmjopen-2020-044566.draft_revisions.pdf]

BMJ Open

BMJ Open is committed to open peer review. As part of this commitment we make the peer review history of every article we publish publicly available.

When an article is published we post the peer reviewers' comments and the authors' responses online. We also post the versions of the paper that were used during peer review. These are the versions that the peer review comments apply to.

The versions of the paper that follow are the versions that were submitted during the peer review process. They are not the versions of record or the final published versions. They should not be cited or distributed as the published version of this manuscript.

BMJ Open is an open access journal and the full, final, typeset and author-corrected version of record of the manuscript is available on our site with no access controls, subscription charges or pay-per-view fees (http://bmjopen.bmj.com).

If you have any questions on BMJ Open's open peer review process please email info.bmjopen@bmj.com

BMJ Open

Utilising recruitment and registry data from England to inform the design of future experimental medicine studies for COVID-19

Journal:	*BMJ Open*
Manuscript ID	bmjopen-2020-044566
Article Type:	Original research
Date Submitted by the Author:	06-Sep-2020

Complete List of Authors:	Cunniffe, Nick; Cambridge University, Department of Clinical Neurosciences<br>Gunter, Simon; Harvard Medical School<br>Brown, Michael; University College London Hospital NHS Trust, Division of Infection<br>Burge, Sarah; University of Cambridge, Cancer Research UK Urological Malignancies Programme, Department of Oncology<br>Coyle, Clare; Hammersmith Hospitals NHS Trust, Department of Cardiology<br>De Soyza, Anthony; Freeman Hospital, Respiratory Medicine<br>Dymond, Tom; Cambridge University Hospitals NHS Foundation Trust, Department of infection and inflammation research<br>Esmail, Hanif; University College London Hospitals NHS Foundation Trust, Hospital for Tropical Diseases; MRC Clinical Trials Unit at UCL<br>Francis, Darrel; Imperial College London, International Centre for Circulatory Health<br>Galloway, Jacqui; Cambridge University Hospitals NHS Foundation Trust, Department of Respiratory Medicine<br>Galloway, James B; Kings College Hospital NHS Foundation Trust; Kings College London, Centre for Rheumatic Diseases<br>Gkrania-Klotsas, Effrossyni; Cambridge University Hospitals NHS Foundation Trust, Department of Infectious Diseases<br>Greenaway, Jane; North Tees General Hospital, Research and Development<br>Katritsis, George; Imperial College London, National Heart and Lung Institute<br>Kanagaratnam, Prapa; Imperial College London<br>Knolle, Martin; Cambridge University Hospitals NHS Foundation Trust, Department of Respiratory Medicine<br>Leonard, Kelly; Cambridge University Hospitals NHS Foundation Trust, Cambridge Urology Translational Research and Clinical Trials Department<br>McIntyre, Zoe; University of Cambridge, School of Clinical medicine, Office for translational research<br>Prudon, Ben; North Tees General Hospital, Department of Respiratory Medicine<br>Rampling, Tommy; Hospital for Tropical Diseases; University College London Hospitals NHS Foundation Trust<br>Torok, Mili; University of Cambridge<br>Warne, Ben; University of Cambridge, Cambridge Institute of

	Therapeutic Immunology and Infectious Disease<br>Yates, Mark; King's College London, Centre for Rheumatic Diseases<br>Matheson, Nicholas; University of Cambridge, Cambridge Insititute for Therapeutic Immunology and Infectious Disease (CITIID), Department of Medicine; NHS Blood and Transplant<br>Su, Li; MRC Biostatistics Unit<br>Villar, Sofia; MRC Biostatistics Unit<br>Stewart, Grant; University of Cambridge, Department of Surgery<br>Toshner, Mark; University of Cambridge, Department of Medicine; NIHR Respiratory Translational Research Collaboration

Keywords:	COVID-19, Clinical trials < THERAPEUTICS, INFECTIOUS DISEASES

SCHOLARONE™
Manuscripts

I, the Submitting Author has the right to grant and does grant on behalf of all authors of the Work (as defined in the below author licence), an exclusive licence and/or a non-exclusive licence for contributions from authors who are: i) UK Crown employees; ii) where BMJ has agreed a CC-BY licence shall apply, and/or iii) in accordance with the terms applicable for US Federal Government officers or employees acting as part of their official duties; on a worldwide, perpetual, irrevocable, royalty-free basis to BMJ Publishing Group Ltd ("BMJ") its licensees and where the relevant Journal is co-owned by BMJ to the co-owners of the Journal, to publish the Work in this journal and any other BMJ products and to exploit all rights, as set out in our licence.

The Submitting Author accepts and understands that any supply made under these terms is made by BMJ to the Submitting Author unless you are acting as an employee on behalf of your employer or a postgraduate student of an affiliated institution which is paying any applicable article publishing charge ("APC") for Open Access articles. Where the Submitting Author wishes to make the Work available on an Open Access basis (and intends to pay the relevant APC), the terms of reuse of such Open Access shall be governed by a Creative Commons licence – details of these licences and which Creative Commons licence will apply to this Work are set out in our licence referred to above.

Other than as permitted in any relevant BMJ Author's Self Archiving Policies, I confirm this Work has not been accepted for publication elsewhere, is not being considered for publication elsewhere and does not duplicate material already published. I confirm all authors consent to publication of this Work and authorise the granting of this licence.

For peer review only

Title page

Title: Utilising recruitment and registry data from England to inform the design of future experimental medicine studies for COVID-19

Authors (in order)

Nick Cunniffe[a] MA MB BChir MRCP[1] <ngc26@cam.ac.uk>

Simon J Gunter[a] MPhil[2] <simon_gunter@hms.harvard.edu>

Michael Brown FRCP PhD DTMH[3] <Michael.brown18@nhs.net>

Sarah Burge PhD[4] <swb35@cam.ac.uk>

Clare Coyle BM BCh BA(Oxon) MRCP[5] <c.coyle@imperial.ac.uk>

Anthony De-Soyza PhD[6] <anthony.de-soyza@newcastle.ac.uk>

Tom Dymond BSc[7] <tom.dymond@addenbrookes.nhs.uk>

Hanif Esmail PhD MRCP FRCPath[8,9,10] <hanif.esmail@nhs.net>

Darrel Francis PhD[11] <darrel@drfrancis.org>

Jacqui Galloway[12] <jacqui.galloway@addenbrookes.nhs.uk>

James Galloway PhD[13] <james.galloway@kcl.ac.uk>

Effrossyni Gkrania-Klotsas MD, PhD[14] <effrossyni.gkrania-klotsas@addenbrookes.nhs.uk>

Jane Greenaway MSc[15] <jane.greenaway@nhs.net>

George Katritsis BSc MBChB MRCP[16] <g.katritsis@imperial.ac.uk>

Prapa Kanagaratnam FRCP PhD[17] <p.kanagaratnam@imperial.ac.uk>

Martin Daniel Knolle MA MB BChir MRCP PhD[12] <martin.knolle@addenbrookes.nhs.uk>

Kelly Leonard BA (Hons)[18] <kelly.leonard@addenbrookes.nhs.uk>

Zoe Catherine McIntyre BSc, MSc[19] <zm276@medschl.cam.ac.uk>

Ben Prudon MBChB(Hons) FRCP Edin[20] <ben.prudon@nhs.net>

For peer review only

Tommy Rampling MRCP DPhil DTMH[21] <Tommy.rampling@nhs.net>

M. Estee Torok PhD FRCP[22] <estee.torok@gmail.com>

Ben Warne MB BChir MRCP[23] <ben.warne@addenbrookes.nhs.uk>

Mark Yates MBChB[13] <mark.yates@kcl.ac.uk>

Nicholas J Matheson PhD MRCP[14,23,24,25] <njm25@cam.ac.uk>

Li Su[26] PhD <li.su@mrc-bsu.cam.ac.uk >

Sofia S. Villar[26] PhD <sofia.villar@mrc-bsu.cam.ac.uk >

Grant D Stewart[b] MBChB PhD[4,27] <gds35@cam.ac.uk>

Mark Toshner*[b] MD FRCP[24,28] <mrt34@medschl.cam.ac.uk>

[a]Co-first author/contributed equally

[b]Joint senior authors/contributed equally

Affiliations

[1]Department of Clinical Neurosciences, University of Cambridge, UK

[2]Harvard Medical School, Harvard University, Boston, MA

[3]Division of Infection, University College London Hospital NHS Trust, UK

[4]Cancer Research UK Urological Malignancies Programme, Department of Oncology, University of Cambridge, UK

[5]Department of Cardiology, Hammersmith Hospital, Imperial College Healthcare NHS Trust, UK

[6]Newcastle University, Newcastle, UK

[7]Department of infection and inflammation research, Cambridge University Hospitals NHS Foundation Trust, UK

[8]Hospital for Tropical Diseases, University College London Hospital NHS Trust, UK

For peer review only

For peer review only - http://bmjopen.bmj.com/site/about/guidelines.xhtml

[9]MRC Clinical Trials Unit, University College London Hospital NHS Trust, UK

[10]Institute for Global Health, University College London, UK

[11]Faculty of Medicine, National Heart & Lung Institute, Imperial College London

[12]Department of Respiratory Medicine, Cambridge University Hospitals NHS Foundation Trust, Cambridge, UK

[13]Centre for Rheumatic Diseases, King's College London, London

[14]Department of Infectious Diseases, Cambridge University Hospitals NHS Foundation Trust, Cambridge, UK

[15]Research & Development, North Tees Hospital, Stockton-on-Tees, UK

[16]Imperial College London, National Heart and Lung Institute, UK

[17]Imperial College, London, UK

[18]Cambridge Urology Translational Research and Clinical Trials Department, Cambridge University Hospitals NHS Foundation Trust, UK

[19]School of Clinical Medicine, Office for Translational Research, University of Cambridge, UK

[20]Department of Respiratory Medicine, North Tees Hospital, Stockton-on-Tees, UK

[21]Division of Pathology, University College London Hospital NHS Trust, UK

[22]University of Cambridge, Cambridge, UK

[23]Cambridge Institute of Therapeutic Immunology and Infectious Disease, University of Cambridge, UK

[24]Department of Medicine, University of Cambridge, UK

[25]NHS Blood and Transplant, Cambridge, UK

[26]MRC Biostatistics Unit, University of Cambridge School of Clinical Medicine, Cambridge Institute of Public Health, UK

[27]Department of Surgery, University of Cambridge, UK

For peer review only - http://bmjopen.bmj.com/site/about/guidelines.xhtml

For peer review only

[28]NIHR Respiratory Translational Research Collaboration, UK

***Corresponding author**

Mark Toshner MD MRCP

University Lecturer

University of Cambridge School of Clinical Medicine

Department of Medicine

Box 157, Addenbrooke's Hospital

Hills Road

Cambridge CB2 0QQ

Tel: 01223 331666

Email: mrt34@medschl.cam.ac.uk

Word count: 2523

For peer review only

Structured abstract

Objectives: To analyse enrolment to interventional trials during the first wave of the coronavirus 2019 (COVID-19) pandemic in England and describe the barriers to successful recruitment in the circumstance of a further wave or future pandemics.

Design: We analysed registered interventional COVID-19 trial data and concurrently did a prospective observational study of hospitalised patients with COVID-19 who were being assessed for eligibility to one of the RECOVERY, C19-ACS or SIMPLE trials.

Setting: Interventional COVID-19 trial data were analysed from the clinicaltrials.gov and ISRCTN databases on July 12, 2020. The patient cohort was taken from 5 centres in a respiratory NIHR network. Population and modelling data were taken from published reports from the UK government and MRC biostatistics unit.

Participants: 2,082 consecutive admitted patients with laboratory-confirmed SARS-CoV-2 infection from March 27, 2020 were included.

Main outcome measures: Proportions enrolled, and reasons for exclusion from the aforementioned trials. Comparisons of trial recruitment targets with estimated feasible recruitment numbers.

Results: Analysis of trial registration data for COVID-19 treatment studies enrolling in England showed that by July 12, 2020, 29,142 participants were needed. In the observational study, 430 (20.7%) proceeded to randomisation. 82 (3.9%) declined participation, 699 (33.6%) were excluded on clinical grounds, 363 (17.4%) were medically fit for discharge, and 153 (7.3%) were receiving palliative care. With 111,037 people hospitalised with COVID-19 in England by July 12, 2020, we determine that 22,985 people were potentially suitable for trial enrolment. We estimate a UK hospitalisation rate of 2.38%, and that another 1.25 million infections would be required to meet recruitment targets of ongoing trials.

For peer review only - http://bmjopen.bmj.com/site/about/guidelines.xhtml

Conclusions: Feasible recruitment rates, study design, and proliferation of trials can limit the number, and size, that will successfully complete recruitment. We consider that fewer, more appropriately designed trials, prioritising cooperation between centres would maximise productivity in a further wave.

For peer review only

For peer review only - http://bmjopen.bmj.com/site/about/guidelines.xhtml

Strengths and limitations of this study

- We comprehensively analysed clinical trial registry data to quantify the number of participants required to successfully complete enrolment to interventional COVID-19 trials based in England in the first wave of the pandemic.
- We simultaneously performed a large, prospective, observational cohort study of 2,082 people hospitalised with COVID-19 to report recruitment rates across a range of secondary and tertiary centres and characterise reasons for trial exclusion.
- Using government data on COVID-19 hospitalisations, we consider the differences between the trials community's aspirations and delivery, and how this might inform our strategy in the event of a second wave.
- Our analysis is limited to data based in England and, while we consider global trials, our conclusions may not be representative of, or readily translatable to, international cohorts.

For peer review only

Introduction

Unless a successful vaccination programme is deployed, the greatest need for coronavirus disease 2019 (COVID-19) remains effective treatments. This presents a substantial challenge. Ostensibly, the response from the experimental medicine community to the first wave has been robust, with more than 1,970 clinical trials planned, recruiting, or completed, at the time of writing.[1] This has enabled enrolment of patients to trials of drugs with known safety profiles – including lopinavir,[2] remdesivir,[3 4] hydroxychloroquine[5 6] and tocilizumab[7] – and led to positive results, such as the 12.1% absolute risk reduction in mortality among ventilated patients treated with dexamethasone.[8]

However, while many of these trials have been pragmatic in terms of selection criteria, the proportion of hospitalised COVID-19 patients being recruited to clinical trials is lower than might have been anticipated; the authors of the RECOVERY trial recently estimated a 10% recruitment rate in the UK.[9] Meanwhile, in areas where public health measures have limited viral transmission, trials have terminated early on account of under recruitment.[10 11] With mounting concern about an ensuing second wave of infection,[12 13] it is increasingly important to learn lessons from the first, and consider the number, size and design of clinical trials that can feasibly be completed.

We hypothesised that the proliferation of SARS-CoV-2 interventional studies during the pandemic and under recognised barriers to recruitment of COVID-19 patients led to unachievable recruitment targets in England. We used data from clinical trial registry databases to quantify recruitment targets and concurrently studied recruitment rates, including reasons for exclusion, across 5 centres enrolling patients at the peak of the first wave of the pandemic. In conjunction with publicly available data from the UK government,

For peer review only - http://bmjopen.bmj.com/site/about/guidelines.xhtml

we consider the differences between the trials community's aspirations and delivery, and how this might inform our strategy if there were a second wave.

For peer review only

For peer review only - http://bmjopen.bmj.com/site/about/guidelines.xhtml

Methods

Establishing recruitment targets for registered trials during first wave

COVID-19 clinical studies registered on clinicaltrials.gov or the International Standard Randomized Controlled Trial Number (ISRCTN) databases were identified and study data downloaded on July 12, 2020. Data for trials based in England, multinational trials with centres in England, and global trials were extracted in turn. Cross-registered studies were identified and accounted for once in the analysis. A manual review determined whether sponsors were academic, non-academic or mixed. Trials were excluded if they were labelled as terminated, withdrawn or suspended. Data for interventional trials examining treatment and prevention were documented, but only trials of COVID-19 treatments were used in the analysis. Analyses were performed using RStudio Version 1.2.5042.

Observational study of recruitment of hospitalised patients

We performed a prospective observational study of 2,082 consecutive patients with SARS-CoV-2 infection at 5 hospitals affiliated to the NIHR-Translational Research Collaboration with representation from secondary and tertiary centres: Cambridge University Hospitals NHS Foundation Trust (CUHFT), Cambridge; Imperial College Healthcare, University College Hospital and King's College Hospitals, London; and University Hospital of North Tees, Middlesbrough. Subjects were admitted and eligibility assessed for: RECOVERY (ISRCTN50189673), C19-ACS (NCT04333407) or SIMPLE (NCT04292730/NCT04292899). CUHFT local R&D approval was undertaken.

Demographic and clinical data were collected by contemporaneous review of potential participants' case notes. A categorical approach subdivided primary reasons subjects were

For peer review only - http://bmjopen.bmj.com/site/about/guidelines.xhtml

not enrolled into: (a) clinical grounds (screening or treating physician judgement that comorbidity or other reason for admission was more critical to patient outcome than COVID-19), (b) medically fit for discharge, (c) receiving end of life care, (d) lack of capacity, (e) patient refusal, (f) interactions with trial drugs, or (g) already on mechanical ventilation. Though already being on mechanical intervention was not an exclusion criterion for RECOVERY, patients categorised as excluded on these grounds were ineligible on account of competing, intensive care-based, studies.

Establishing feasible recruitment for registered trials during first wave

Using publicly available UK government data of the numbers of patients with COVID-19 admitted to English hospitals during the first wave between March 17 and August 5, 2020,[14] and the recruitment rate (with 95% confidence interval (CI) for one sample proportion with continuity correction) from the aforementioned observational study, we estimated a maximum bound for the accumulated feasible recruitment during that time. Simultaneously, we used the estimated cumulative number of infected cases in England by 12 July provided by MRC Biostatistics Unit at the University of Cambridge[15] to calculate an approximate hospitalisation rate in England among COVID-19 infections. We based our estimates on data from centres in England as the infection rate estimates were more reliable, hospitalisation criteria were different in Wales,[14] and the 5 hospitals included in this study are all from England.

Patient and public involvement

This was a time-critical study in response to a Public Health Emergency of International Concern. Patients or the public were not involved in the design, conduct, or reporting of this research.

For peer review only - http://bmjopen.bmj.com/site/about/guidelines.xhtml

For peer review only

For peer review only - http://bmjopen.bmj.com/site/about/guidelines.xhtml

Results

Establishing recruitment targets to registered trials during first wave

Clinical trial registry data were downloaded on July 12, 2020; 28 interventional studies were included in our analysis of those registered in England. 22 (78%) were academically sponsored, 5 (18%) were non-academically sponsored and 1 (4%) was mixed. The first registration date of a COVID-19 treatment trial in England was March 22; the earliest registered start date was March 12. Analysis of recruitment targets for each trial revealed that 46,154 participants would be required to complete recruitment to all studies in England (Table 1): 17,012 people are required for trials of prophylactic drugs to prevent COVID-19, while 29,142 are needed for those treating established COVID-19 (Table 1). The median (IQR) treatment trial recruitment target was 195 (50-793).

By contrast, the global situation is such that 1,107 registered interventional trials were ongoing or completed, requiring 566,872 patients to be randomised to allow their completion; 306,426 of these are needed for trials of COVID-19 treatments (Figure 1A and 1B). These trials are geographically clustered in China, North America and Europe (Figure 1C).

Observational study of clinical trial enrolment

From March 27 to May 22, 2020 a total of 2,082 consecutive patients were included across the 5 sites (Table 2). Age and sex data were available for 1,971 patients: the median (IQR) age was 71 (58-82) and 56.2% were male. Across the four trials, 430 (20.7%, 95% CI [18.95%, 22.47%]) proceeded to randomisation.

Of the remaining 1,652 patients, 82 (3.9%) declined participation, 363 (17.4%) were medically fit for discharge, 153 (7.3%) were receiving end of life care and 106 (5.1%) were

For peer review only - http://bmjopen.bmj.com/site/about/guidelines.xhtml

mechanically ventilated at the time of screening. In 699 (33.6%) patients, the screening or treating physician determined that the potential participant should not be enrolled on account of clinical grounds or trial exclusion criteria.

Establishing feasible recruitment for registered trials during first wave

By combining these observed recruitment rates with publicly reported hospitalisation data (between March 17, and July 12, 2020), we estimated a maximum upper bound for the accumulated feasible recruitment for registered trials of COVID-19 treatments in England during the first wave (Figure 2).

The estimated number of cumulative infected cases by 12 July reported by MRC Biostatistics Unit is 4.67 million with a 95% credible interval [3.76, 6.04]. Combined with the number of cumulative admitted patients in England by 12 July from government data (i.e. 111,037 hospital admissions), this gives an approximate hospitalisation rate 2.38% [1.84%, 2.95%] in England during the first wave.

Our analysis indicates that by July 12th, 6,158 patients might still be needed to meet the total recruitment targets for currently recruiting clinical trials. If considering uncertainty in recruitment rate estimate reflected by 95% CI [18.95%, 22.47%], 4,192-8,100 patients might be required to meet recruitment target. Assuming the recruitment rate 20.7%, this implies that 29,749 hospitalised patients would need to be screened for these trials to complete recruitment. With the approximate hospitalisation rate 2.38% in England as observed in the first wave, this would require 1.249 million patients to be infected.

For peer review only - http://bmjopen.bmj.com/site/about/guidelines.xhtml

With the daily infection rate for UK estimated to be 3,310 (95% credible interval [2440, 4460]) on 12 July,[15] it is highly unlikely such a large number of hospitalisations would occur unless there is an increase in the infection numbers (or a second wave). Indeed, incorporating hospitalisation data to August 5, 2020, shows minimal progress toward the recruitment target, assuming no new trials were approved after July 12, 2020 (Figure 2B).

For peer review only

For peer review only - http://bmjopen.bmj.com/site/about/guidelines.xhtml

Discussion

We found that the proliferation of clinical trials[1] in response to the first wave of the COVID-19 pandemic in England required 29,142 participants to complete enrolment to those registered with a trials database. Globally, 306,426 participants are required to meet recruitment targets for trials of treatments of COVID-19. Meanwhile, in our multicentre prospective observational cohort study of patients admitted to hospital with laboratory-confirmed COVID-19, 79.3% of potential participants were not recruited to a clinical trial; the reasons for excluding patients were varied and clarify the challenges faced in both general hospitals and well-resourced centres experienced in experimental medicine. Our experience is consistent with the general literature on clinical trial recruitment where many factors have been posited to contribute to heterogeneity of recruitment.[16] With 111,037 people hospitalised in England between March 17 and July 12, 2020, our net recruitment rate suggests that 22,985 (21,042-24,950 if taking into account uncertainty in recruitment rate estimate by random errors) would have been potentially suitable for selection in the first wave. However, this is clearly an overestimate, given that it would require each of these individuals to be hospitalised in geographical locations where medical centres were undertaking these trials. In the first wave, most general clinical trials infrastructure was mothballed for normal activity and therefore easily seconded towards COVID-19 and this may not be the case in subsequent "waves". It must also be recalled that most recruitment in the first wave was undertaken as hospitals were actively reconfiguring services. A stable hospital infrastructure may positively impact on ease of delivery in the future. Nevertheless, unless there is a second wave it is highly unlikely that the total recruitment target will be met in any reasonable timeframe.

For peer review only - http://bmjopen.bmj.com/site/about/guidelines.xhtml

Strengths of our study are that our analyses of registry and population databases utilised the largest and most robust data available. Meanwhile, our observational study applied a large cohort size, prospective data acquisition, and recorded detailed reasons for excluding patients. By using both secondary and tertiary care centres, we believe our results are generalisable to other hospitals in the UK. Also, by following studies with minimal selection criteria, particularly in the RECOVERY trial, we reduced the chance of underestimating trial recruitment. Our study does have limitations. First, our predictions were based on registry data for studies based in England alone; we did not include the numbers of participants required to be recruited into multinational trials in which the English centres were involved. The result is that we have likely underestimated the trial recruitment target for England and, by extension, the gap between this and the number of participants available. Second, although we used hospitalisation data from 17 March 2020, as this was the time the UK government commenced public reporting of COVID-19 admissions, all trials included in our registry analysis were not recruiting at that stage; the earliest start date for a trial registered in England was March 12, 2020, but the last trial start date was not until July 7, 2020. In this sense, using cumulative number of admitted patients in our prediction is optimistic. Third, we only included the two registry datasets in most widespread use, and so may have further underestimated the number of studies and participants required. Fourth, the 95% CI for recruitment rate estimate only reflects the uncertainty due to random errors in the data, it does not consider the uncertainty due to unrepresentativeness of data from the 5 hospital centres in our study. Finally, although we illustrate the scale of trial recruitment required globally, the populations tested may not be representative of, or translatable to, international cohorts.

Our study is the first to characterise the suitability and barriers for trial enrolment for a complete cohort of hospitalised patients with COVID-19. Results of trials published to date

For peer review only - http://bmjopen.bmj.com/site/about/guidelines.xhtml

convey a different message: interventional studies of lopinavir and remdesivir, for example, have recruitment rates ranging from 55.7%-96.0%.[2-4] This difference is most likely explained by the different denominators used in our calculations: the consort diagrams in clinical trials are unlikely to include every single patient hospitalised with a positive test. Instead, our results align with or exceed other centres, such as the 10% recruitment rate to RECOVERY.[9] During the 2013-16 Ebola Virus Disease (EVD) epidemic in west Africa, most clinical trials during that crisis either started too late to enrol sufficient case numbers or were simply unable to reach their recruitment targets.[17] Our study shows that trials in England started recruiting relatively quickly, however many are highly unlikely to recruit on time; we conclude that starting early is important but not enough to ensure recruitment targets are met. Finally, it is notable that our calculated hospitalisation rate of 2.38% is lower than that observed in Wuhan,[18] which if applied to the UK age structure,[19] is equivalent to approximately 5.8%.

The disparity between the realistic recruitment rates and high requirements we report leads us to conclude that the scientific community should be increasingly selective in the number, size and design of clinical trials deployed in the COVID-19 pandemic; our findings have meaning for those planning single trials, and those strategizing the national response. Potential solutions include practical changes to trial design, for instance capturing patients earlier in their disease path, and adopting dynamic and adaptive trial designs.[20] Yet, such measures are unlikely to bridge the currently estimated large recruitment gap. Instead, it may be necessary for healthcare authorities and policy makers to foster more academic cooperation to prioritise compounds, prevent duplication and, perhaps more radically, perform real-time meta-analyses of ongoing trials of the same therapies and provide stop/go recommendations across trials to rationalise treatment and prevent multiple studies delaying reporting.[21] Indeed, proposals have been forthcoming for mechanisms by which data from different trials might

For peer review only - http://bmjopen.bmj.com/site/about/guidelines.xhtml

For peer review only

be shared and analysed in a robust and scientifically meaningful way.[22] These conclusions are not dissimilar to reflections from the Ebola pandemic, when there was a strong call for strengthening and coordinating research efforts in response to outbreaks of emerging infectious diseases.[23 24] For planning future trials and deriving realistic recruitment targets, real-time tracking of the pandemic, as data accumulate over time, is essential to plan research in response of an emerging epidemics outbreak. The Medical Research Council (MRC) Biostatistics Unit regularly nowcast and forecast COVID-19 infections and deaths.[15] This information feeds directly to SAGE sub-group, Scientific Pandemic Influenza sub-group on Modelling (SPI-M) and to regional PHE teams. This same data could be used to establish realistic recruitment trends to inform, monitor and coordinate research efforts both for treatment and prevention trials.

Multiple questions remain for future research. In particular, it remains unclear how relaxing of non-pharmacological interventions will affect transmission rates, and therefore the achievability of remaining recruitment to these trials. It is also unknown how a second wave would evolve, and whether more or fewer people will develop the illness than was seen in the first. Nonetheless, we conclude that clinical trialists and healthcare authorities must consider the recruitment challenges when determining the feasibility of clinical trials in a second wave and urgently rationalise those currently active.

For peer review only - http://bmjopen.bmj.com/site/about/guidelines.xhtml

Acknowledgements

We acknowledge the use of original data from the UK government (https://coronavirus.data.gov.uk/) and infection case estimates of England in the nowcasting and forecasting report provided by the MRC Biostatistics Unit.

Footnotes

Contributors: Dr Cunniffe and Dr Toshner accept full responsibility for the work and conduct of the study, had access to the data, and controlled the decision to publish. Dr Cunniffe and Simon Gunter contributed equally as co-first authors. Dr Toshner and Prof Stewart contributed equally as joint senior authors.

Study concept and design: Cunniffe, Gunter, Stewart, Su, Villar, Toshner

Acquisition, analysis or interpretation of data: Cunniffe, Gunter, Stewart, Su, Villar, Toshner, Brown, Burge, Coyle, De-Soyza, Dymond, Esmail, Francis, Jacqui Galloway, James Galloway, Gkrania-Klotsas, Greenaway, Katritsis, Kanagaratnam, Knolle, Leonard, McIntyre, Prudon, Rampling, Torok, Warne, Yates and Matheson

Drafting of manuscript: Cunniffe, Gunter, Stewart, Su, Villar, Toshner

Critical revision of the manuscript: Cunniffe, Gunter, Stewart, Su, Villar, Toshner, Brown, Burge, Coyle, De-Soyza, Dymond, Esmail, Francis, Jacqui Galloway James Galloway PhD, Gkrania-Klotsas, Greenaway, Katritsis, Kanagaratnam, Knolle, Leonard, McIntyre, Prudon, Rampling, Torok, Warne and Matheson

Statistical analysis: Cunniffe, Gunter, Warne, Matheson

Competing interests: None declared.

For peer review only - http://bmjopen.bmj.com/site/about/guidelines.xhtml

Transparency statement: Dr Toshner, the manuscript's guarantor, affirms that the manuscript is an honest, accurate, and transparent account of the study being reported; that no important aspects of the study have been omitted; and that any discrepancies from the study as originally planned (and, if relevant, registered) have been explained.

Funding: Dr Burge and Prof Stewart declare funding from a Cancer Research UK Major Centre award (C9685/A25117). Dr Coyle declares funding from the British Heart Foundation. Dr Gkrania-Klotsas is supported by an NIHR Greenshoots personal Award from the Clinical Research Network. Prof Kanagaratnam declares funding from the Imperial College Covid 19 Research Fund. Dr Knolle declares funding from MRC CARP. Dr Torok declares support from a Clinician Scientist Fellowship (Academy of Medical and the Health Foundation) and the NIHR Cambridge Biomedical Research Centre. Dr Warne receives funding from the National Institute for Health Research Cambridge Biomedical Research Centre at the Cambridge University Hospitals NHS Foundation Trust. Dr Yates' salary is funded by The British Society for Rheumatology and Versus Arthritis. Dr Matheson declares funding from MRC (CSF MR/P008801/1) and NHSBT (WPA15-02). Dr Toshner is funded by the NIHR Cambridge Biomedical Research Centre. This work was enabled by co-operation of the NIHR Respiratory Translational Research Collaboration.

For peer review only

Ethical approval: Data were acquired within the ethical approvals of the aforementioned trials.

Data sharing: All relevant data is included in the manuscript. Data from the clinical trials registries, UK government, and MRC biostatistics unit are available on public websites.

For peer review only - http://bmjopen.bmj.com/site/about/guidelines.xhtml

For peer review only

For peer review only - http://bmjopen.bmj.com/site/about/guidelines.xhtml

Figure legends

Figure 1. The proliferation of global clinical trials in response to COVID-19. A: cumulative number of enrolling studies registered with clinicaltrials.gov or ISRCTN until Jul 12, 2020, subdivided by those testing drugs for COVID-19 treatment and prevention. B: cumulative number of participants required to meet recruitment targets for registered clinical trials. C: geographical distribution of COVID-19 clinical trials.

Figure 2. Feasibility of achieving target recruitment in England for COVID-19 interventional studies. A: cumulative number of enrolling studies in England registered with clinicaltrials.gov or ISRCTN until July 12, 2020, subdivided by those testing drugs for COVID-19 treatment and prevention. B: cumulative number of participants required to meet recruitment targets for registered COVID-19 treatment trials until July 12, 2020, and predicted number of patients whom would have been eligible for randomisation (grey shaded area represents point-wise 95% confidence band for the predictive cumulative number of eligible patients using the lower and upper value of 95% confidence interval for the recruitment rate estimate with continuity correction). The reduction in the infection rate in England means that the recruitment target at July 12 is unlikely to be reached unless there is a second wave; further illustrated by extending hospitalisation data to August 5, 2020.

For peer review only

For peer review only - http://bmjopen.bmj.com/site/about/guidelines.xhtml

Tables

	Number of Trials	Number of Participants
**Global Trials**		
Prevention	172	260,446
Treatment	935	306,426
Total	1,107	566,872
**UK Multi-National and National Trials**		
Prevention	11	97,272
Treatment	38	44,362
Total	49	141,634
**England Trials**		
Prevention	8	17,012
Treatment	20	29,142
Total	28	46,154

Table 1: Summary of number of trials and required numbers of participants

For peer review only

	RECOVERY				COMBINATION*	C19-ACS	SIMPLE	**Total**
**Total screened per centre**	281	83	415	Total (779)	445	784	74	2,082
**Number recruited (%)**	35 (12.5)	16 (19.3)	185 (44.6)	236 (30.3)	124 (27.9)	56 (7.1)	14 (18.9)	**430 (20.7)**
Refused participation (%)	10 (3.6)	19 (22.9)	16 (3.9)	45 (5.8)	8 (1.8)	29 (3.7)	0 (0.0)	82 (3.9)
Clinical grounds/trial exclusion criteria(%)	83 (29.5)	15 (18.1)	40 (9.6)	138 (17.7)	167 (37.5)	365 (46.6)	29 (39.2)	699 (33.6)
Lacked capacity (%)	22 (7.8)	0 (0.0)	1 (0.2)	23 (3.0)	16 (3.6)	98 (12.5)	0 (0.0)	137 (6.6)
Mechanical ventilation (%)	37 (13.2)	7 (8.4)	0 (0.0)	44 (5.6)	7 (1.6)	48 (6.1)	7 (9.5)	106 (5.1)
Drug interactions (%)	12 (4.3)	2 (2.4)	0 (0.0)	14 (1.8)	2 (0.4)	1 (0.1)	0 (0.0)	17 (0.8)
Medically fit for discharge (%)	55 (19.6)	14 (16.9)	77 (18.6)	146 (18.7)	65 (14.6)	136 (17.3)	16 (21.6)	363 (17.4)
Palliative care (%)	19 (6.8)	7 (8.4)	61 (14.7)	87 (11.2)	8 (1.8)	51 (6.5)	7 (9.5)	153 (7.3)
Not approached or considered (%)	8 (2.8)	3 (3.6)	35 (8.4)	46 (5.9)	48 (10.8)	0 (0.0)	1 (1.4)	95 (4.6)
**Total not recruited (%)**	246 (87.5)	67 (80.7)	230 (55.4)	543 (69.7)	321 (72.1)	728 (92.9)	60 (81.1)	**1,652 (79.3)**

Table 2: screening data for 2,082 consecutive patients with laboratory-confirmed SARS-CoV-2 admitted to one of 5 centres. *centre screened concurrently to both RECOVERY and SIMPLE: moderate and severe trials.

For peer review only

References

1. Thorlund K, Dron L, Park J, et al. A real-time dashboard of clinical trials for COVID-19. *The Lancet Digital Health* 2020;2(6):e286-e87. doi: 10.1016/S2589-7500(20)30086-8

2. Cao B, Wang Y, Wen D, et al. A Trial of Lopinavir–Ritonavir in Adults Hospitalized with Severe Covid-19. *New England Journal of Medicine* 2020;382(19):1787-99. doi: 10.1056/NEJMoa2001282

3. Beigel JH, Tomashek KM, Dodd LE, et al. Remdesivir for the Treatment of Covid-19 — Preliminary Report. *New England Journal of Medicine* 2020 doi: 10.1056/NEJMoa2007764

4. Wang Y, Zhang D, Du G, et al. Remdesivir in adults with severe COVID-19: a randomised, double-blind, placebo-controlled, multicentre trial. *The Lancet* 2020;395(10236):1569-78. doi: 10.1016/S0140-6736(20)31022-9

5. Geleris J, Sun Y, Platt J, et al. Observational Study of Hydroxychloroquine in Hospitalized Patients with Covid-19. *New England Journal of Medicine* 2020;382(25):2411-18. doi: 10.1056/NEJMoa2012410

6. Gautret P, Lagier J-C, Parola P, et al. Hydroxychloroquine and azithromycin as a treatment of COVID-19: results of an open-label non-randomized clinical trial. *International Journal of Antimicrobial Agents* 2020:105949. doi: https://doi.org/10.1016/j.ijantimicag.2020.105949

7. Zhang C, Wu Z, Li J-W, et al. Cytokine release syndrome in severe COVID-19: interleukin-6 receptor antagonist tocilizumab may be the key to reduce mortality. *International Journal of Antimicrobial Agents* 2020;55(5):105954. doi: https://doi.org/10.1016/j.ijantimicag.2020.105954

For peer review only

8. The RECOVERY Collaborative Group. Dexamethasone in Hospitalized Patients with Covid-19 — Preliminary Report. *New England Journal of Medicine* 2020 doi: 10.1056/NEJMoa2021436

9. Wilkinson E. RECOVERY trial: the UK covid-19 study resetting expectations for clinical trials. *BMJ* 2020;369:m1626. doi: 10.1136/bmj.m1626

10. Norrie JD. Remdesivir for COVID-19: challenges of underpowered studies. *The Lancet* 2020;395(10236):1525-27. doi: 10.1016/S0140-6736(20)31023-0

11. Li L, Zhang W, Hu Y, et al. Effect of Convalescent Plasma Therapy on Time to Clinical Improvement in Patients With Severe and Life-threatening COVID-19: A Randomized Clinical Trial. *JAMA* 2020;324(5):460-70. doi: 10.1001/jama.2020.10044

12. Xu S, Li Y. Beware of the second wave of COVID-19. *The Lancet* 2020;395(10233):1321-22. doi: 10.1016/S0140-6736(20)30845-X

13. Petersen E, Koopmans M, Go U, et al. Comparing SARS-CoV-2 with SARS-CoV and influenza pandemics. *The Lancet Infectious Diseases* doi: 10.1016/S1473-3099(20)30484-9

14. Publich Health England. Coronavirus (COVID-19) in the UK 2020 [Available from: https://coronavirus.data.gov.uk/healthcare] accessed August 5 2020.

15. MRC Biostatistics Unit. COVID-19: nowcast and forecast 2020 [Available from: https://www.mrc-bsu.cam.ac.uk/now-casting/report-on-nowcasting-and-forecasting-6th-august-2020/] accessed August 14 2020.

16. Thoma A, Farrokhyar F, McKnight L, et al. Practical tips for surgical research: how to optimize patient recruitment. *Can J Surg* 2010;53(3):205-10. [published Online First: 2010/05/29]

For peer review only - http://bmjopen.bmj.com/site/about/guidelines.xhtml

17. Rojek A, Horby P, Dunning J. Insights from clinical research completed during the west Africa Ebola virus disease epidemic. *The Lancet Infectious Diseases* 2017;17(9):e280-e92. doi: 10.1016/S1473-3099(17)30234-7

18. Verity R, Okell LC, Dorigatti I, et al. Estimates of the severity of coronavirus disease 2019: a model-based analysis. *The Lancet Infectious Diseases* 2020;20(6):669-77. doi: 10.1016/S1473-3099(20)30243-7

19. Office of national statistics. Old age structure variant - UK population in age groups 2018 [Available from: https://www.ons.gov.uk/peoplepopulationandcommunity/populationandmigration/populationprojections/datasets/tablel21oldagestructurevariantukpopulationinagegroups] accessed May, 28 2020.

20. Brueckner M, Titman A, Jaki T, et al. Performance of different clinical trial designs to evaluate treatments during an epidemic. *PLOS ONE* 2018;13(9):e0203387. doi: 10.1371/journal.pone.0203387

21. Bauchner H, Fontanarosa PB. Randomized Clinical Trials and COVID-19: Managing Expectations. *JAMA* 2020;323(22):2262-63. doi: 10.1001/jama.2020.8115

22. Petkova E, Antman EM, Troxel AB. Pooling Data From Individual Clinical Trials in the COVID-19 Era. *JAMA* 2020 doi: 10.1001/jama.2020.13042

23. World Health Organisation. Report of the Ebola Interim Assessment Panel - May 2015, 2015.

24. National Academies of Sciences E, Medicine. Integrating Clinical Research into Epidemic Response: The Ebola Experience. Washington, DC: The National Academies Press 2017.

For peer review only

For peer review only - http://bmjopen.bmj.com/site/about/guidelines.xhtml

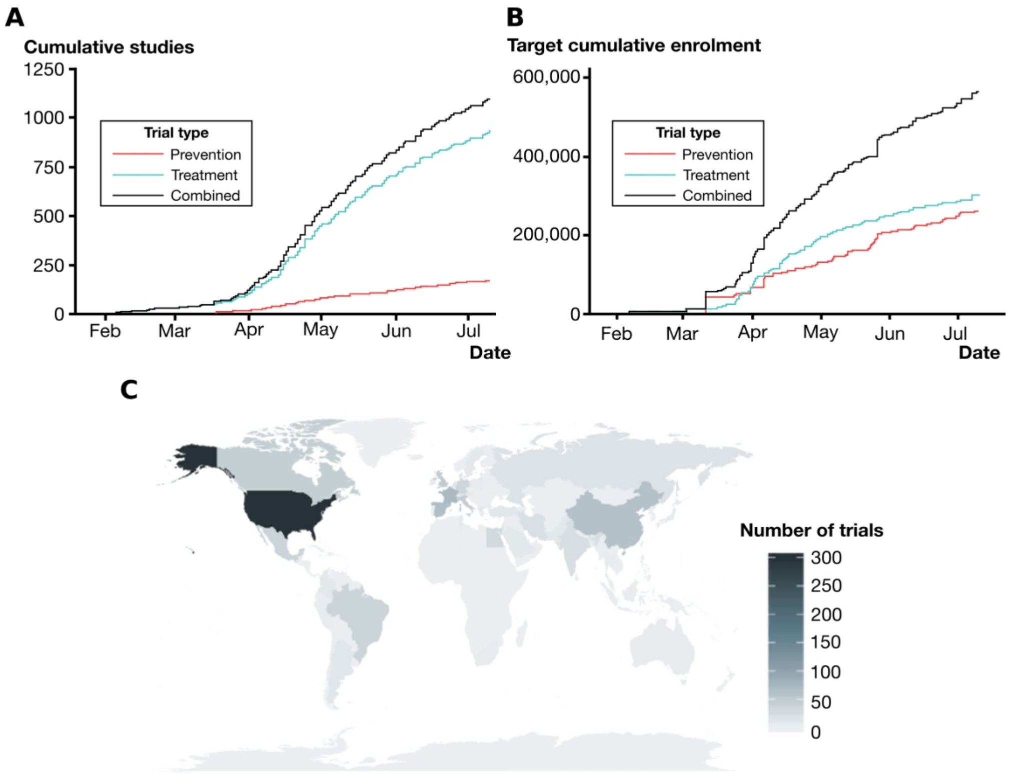

Figure 1. The proliferation of global clinical trials in response to COVID-19. A: cumulative number of enrolling studies registered with clinicaltrials.gov or ISRCTN until Jul 12, 2020, subdivided by those testing drugs for COVID-19 treatment and prevention. B: cumulative number of participants required to meet recruitment targets for registered clinical trials. C: geographical distribution of COVID-19 clinical trials.

193x153mm (300 x 300 DPI)

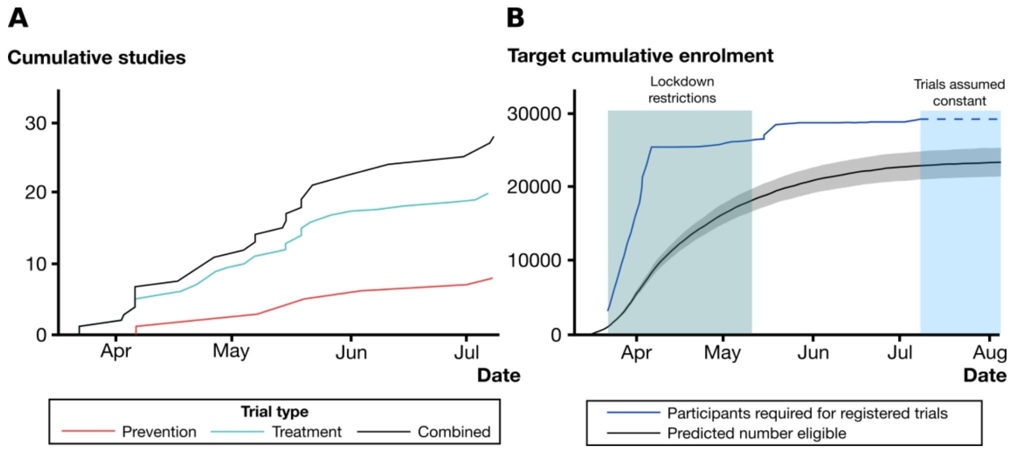

Figure 2. Feasibility of achieving target recruitment in England for COVID-19 interventional studies. A: cumulative number of enrolling studies in England registered with clinicaltrials.gov or ISRCTN until July 12, 2020, subdivided by those testing drugs for COVID-19 treatment and prevention. B: cumulative number of participants required to meet recruitment targets for registered COVID-19 treatment trials until July 12, 2020, and predicted number of patients whom would have been eligible for randomisation (grey shaded area represents point-wise 95% confidence band for the predictive cumulative number of eligible patients using the lower and upper value of 95% confidence interval for the recruitment rate estimate with continuity correction). The reduction in the infection rate in England means that the recruitment target at July 12 is unlikely to be reached unless there is a second wave; further illustrated by extending hospitalisation data to August 5, 2020.

184x80mm (300 x 300 DPI)

STROBE Statement—Checklist of items that should be included in reports of ***cohort studies***

	Item No	Recommendation	Page No
**Title and abstract**	1	(*a*) Indicate the study's design with a commonly used term in the title or the abstract<br>(*b*) Provide in the abstract an informative and balanced summary of what was done and what was found	1,5
**Introduction**			
Background/rationale	2	Explain the scientific background and rationale for the investigation being reported	8,9
Objectives	3	State specific objectives, including any prespecified hypotheses	5,8,9
**Methods**			
Study design	4	Present key elements of study design early in the paper	5,8,9
Setting	5	Describe the setting, locations, and relevant dates, including periods of recruitment, exposure, follow-up, and data collection	5,10,11
Participants	6	(*a*) Give the eligibility criteria, and the sources and methods of selection of participants. Describe methods of follow-up<br>(*b*) For matched studies, give matching criteria and number of exposed and unexposed	5,10,11
Variables	7	Clearly define all outcomes, exposures, predictors, potential confounders, and effect modifiers. Give diagnostic criteria, if applicable	5,10,11
Data sources/ measurement	8*	For each variable of interest, give sources of data and details of methods of assessment (measurement). Describe comparability of assessment methods if there is more than one group	5,10,11
Bias	9	Describe any efforts to address potential sources of bias	10,11
Study size	10	Explain how the study size was arrived at	10,11
Quantitative variables	11	Explain how quantitative variables were handled in the analyses. If applicable, describe which groupings were chosen and why	10,11
Statistical methods	12	(*a*) Describe all statistical methods, including those used to control for confounding<br>(*b*) Describe any methods used to examine subgroups and interactions<br>(*c*) Explain how missing data were addressed<br>(*d*) If applicable, explain how loss to follow-up was addressed<br>(*e*) Describe any sensitivity analyses	10,11
**Results**			
Participants	13*	(a) Report numbers of individuals at each stage of study—eg numbers potentially eligible, examined for eligibility, confirmed eligible, included in the study, completing follow-up, and analysed<br>(b) Give reasons for non-participation at each stage<br>(c) Consider use of a flow diagram	5,13
Descriptive data	14*	(a) Give characteristics of study participants (eg demographic, clinical, social) and information on exposures and potential confounders<br>(b) Indicate number of participants with missing data for each variable of interest<br>(c) Summarise follow-up time (eg, average and total amount)	5,13
Outcome data	15*	Report numbers of outcome events or summary measures over time	5,13,14,15

For peer review only

Main results	16	(*a*) Give unadjusted estimates and, if applicable, confounder-adjusted estimates and their precision (eg, 95% confidence interval). Make clear which confounders were adjusted for and why they were included<br>(*b*) Report category boundaries when continuous variables were categorized<br>(*c*) If relevant, consider translating estimates of relative risk into absolute risk for a meaningful time period	13-15
Other analyses	17	Report other analyses done—eg analyses of subgroups and interactions, and sensitivity analyses	14-15
**Discussion**			
Key results	18	Summarise key results with reference to study objectives	16
Limitations	19	Discuss limitations of the study, taking into account sources of potential bias or imprecision. Discuss both direction and magnitude of any potential bias	18
Interpretation	20	Give a cautious overall interpretation of results considering objectives, limitations, multiplicity of analyses, results from similar studies, and other relevant evidence	17,18,19
Generalisability	21	Discuss the generalisability (external validity) of the study results	16,17,18
**Other information**			
Funding	22	Give the source of funding and the role of the funders for the present study and, if applicable, for the original study on which the present article is based	22

*Give information separately for exposed and unexposed groups.

Note: An Explanation and Elaboration article discusses each checklist item and gives methodological background and published examples of transparent reporting. The STROBE checklist is best used in conjunction with this article (freely available on the Web sites of PLoS Medicine at http://www.plosmedicine.org/, Annals of Internal Medicine at http://www.annals.org/, and Epidemiology at http://www.epidem.com/). Information on the STROBE Initiative is available at http://www.strobe-statement.org.

For peer review only

BMJ Open

How achievable are COVID-19 clinical trial recruitment targets? A UK observational cohort study and trials registry analysis

Journal:	*BMJ Open*
Manuscript ID	bmjopen-2020-044566.R1
Article Type:	Original research
Date Submitted by the Author:	16-Sep-2020
Complete List of Authors:	Cunniffe, Nick; Cambridge University, Department of Clinical Neurosciences<br>Gunter, Simon; Harvard Medical School<br>Brown, Michael; University College London Hospital NHS Trust, Division of Infection<br>Burge, Sarah; University of Cambridge, Cancer Research UK Urological Malignancies Programme, Department of Oncology<br>Coyle, Clare; Hammersmith Hospitals NHS Trust, Department of Cardiology<br>De Soyza, Anthony; Freeman Hospital, Respiratory Medicine<br>Dymond, Tom; Cambridge University Hospitals NHS Foundation Trust, Department of infection and inflammation research<br>Esmail, Hanif; University College London Hospitals NHS Foundation Trust, Hospital for Tropical Diseases; MRC Clinical Trials Unit at UCL<br>Francis, Darrel; Imperial College London, International Centre for Circulatory Health<br>Galloway, Jacqui; Cambridge University Hospitals NHS Foundation Trust, Department of Respiratory Medicine<br>Galloway, James B; Kings College Hospital NHS Foundation Trust; Kings College London, Centre for Rheumatic Diseases<br>Gkrania-Klotsas, Effrossyni; Cambridge University Hospitals NHS Foundation Trust, Department of Infectious Diseases<br>Greenaway, Jane; North Tees General Hospital, Research and Development<br>Katritsis, George; Imperial College London, National Heart and Lung Institute<br>Kanagaratnam, Prapa; Imperial College London<br>Knolle, Martin; Cambridge University Hospitals NHS Foundation Trust, Department of Respiratory Medicine<br>Leonard, Kelly; Cambridge University Hospitals NHS Foundation Trust, Cambridge Urology Translational Research and Clinical Trials Department<br>McIntyre, Zoe; University of Cambridge, School of Clinical medicine, Office for translational research<br>Prudon, Ben; North Tees General Hospital, Department of Respiratory Medicine<br>Rampling, Tommy; Hospital for Tropical Diseases; University College London Hospitals NHS Foundation Trust<br>Torok, Mili; University of Cambridge<br>Warne, Ben; University of Cambridge, Cambridge Institute of

	Therapeutic Immunology and Infectious Disease<br>Yates, Mark; King's College London, Centre for Rheumatic Diseases<br>Matheson, Nicholas; University of Cambridge, Cambridge Insititute for Therapeutic Immunology and Infectious Disease (CITIID), Department of Medicine; NHS Blood and Transplant<br>Su, Li; MRC Biostatistics Unit<br>Villar, Sofia; MRC Biostatistics Unit<br>Stewart, Grant; University of Cambridge, Department of Surgery<br>Toshner, Mark; University of Cambridge, Department of Medicine; NIHR Respiratory Translational Research Collaboration
**Primary Subject Heading**:	Infectious diseases
Secondary Subject Heading:	Respiratory medicine, Health policy
Keywords:	COVID-19, Clinical trials < THERAPEUTICS, INFECTIOUS DISEASES

I, the Submitting Author has the right to grant and does grant on behalf of all authors of the Work (as defined in the below author licence), an exclusive licence and/or a non-exclusive licence for contributions from authors who are: i) UK Crown employees; ii) where BMJ has agreed a CC-BY licence shall apply, and/or iii) in accordance with the terms applicable for US Federal Government officers or employees acting as part of their official duties; on a worldwide, perpetual, irrevocable, royalty-free basis to BMJ Publishing Group Ltd ("BMJ") its licensees and where the relevant Journal is co-owned by BMJ to the co-owners of the Journal, to publish the Work in this journal and any other BMJ products and to exploit all rights, as set out in our licence.

The Submitting Author accepts and understands that any supply made under these terms is made by BMJ to the Submitting Author unless you are acting as an employee on behalf of your employer or a postgraduate student of an affiliated institution which is paying any applicable article publishing charge ("APC") for Open Access articles. Where the Submitting Author wishes to make the Work available on an Open Access basis (and intends to pay the relevant APC), the terms of reuse of such Open Access shall be governed by a Creative Commons licence – details of these licences and which Creative Commons licence will apply to this Work are set out in our licence referred to above.

Other than as permitted in any relevant BMJ Author's Self Archiving Policies, I confirm this Work has not been accepted for publication elsewhere, is not being considered for publication elsewhere and does not duplicate material already published. I confirm all authors consent to publication of this Work and authorise the granting of this licence.

For peer review only

Title page

Title: How achievable are COVID-19 clinical trial recruitment targets? A UK observational cohort study and trials registry analysis

Authors (in order)

Nick Cunniffe[a] MA MB BChir MRCP[1] <ngc26@cam.ac.uk>

Simon J Gunter[a] MPhil[2] <simon_gunter@hms.harvard.edu>

Michael Brown FRCP PhD DTMH[3] <Michael.brown18@nhs.net>

Sarah Burge PhD[4] <swb35@cam.ac.uk>

Clare Coyle BM BCh BA(Oxon) MRCP[5] <c.coyle@imperial.ac.uk>

Anthony De-Soyza PhD[6] <anthony.de-soyza@newcastle.ac.uk>

Tom Dymond BSc[7] <tom.dymond@addenbrookes.nhs.uk>

Hanif Esmail PhD MRCP FRCPath[8,9,10] <hanif.esmail@nhs.net>

Darrel Francis PhD[11] <darrel@drfrancis.org>

Jacqui Galloway[12] <jacqui.galloway@addenbrookes.nhs.uk>

James Galloway PhD[13] <james.galloway@kcl.ac.uk>

Effrossyni Gkrania-Klotsas MD, PhD[14] <effrossyni.gkrania-klotsas@addenbrookes.nhs.uk>

Jane Greenaway MSc[15] <jane.greenaway@nhs.net>

George Katritsis BSc MBChB MRCP[16] <g.katritsis@imperial.ac.uk>

Prapa Kanagaratnam FRCP PhD[17] <p.kanagaratnam@imperial.ac.uk>

Martin Daniel Knolle MA MB BChir MRCP PhD[12] <martin.knolle@addenbrookes.nhs.uk>

Kelly Leonard BA (Hons)[18] <kelly.leonard@addenbrookes.nhs.uk>

Zoe Catherine McIntyre BSc, MSc[19] <zm276@medschl.cam.ac.uk>

Ben Prudon MBChB(Hons) FRCP Edin[20] <ben.prudon@nhs.net>

For peer review only

Tommy Rampling MRCP DPhil DTMH[21] <Tommy.rampling@nhs.net>

M. Estee Torok PhD FRCP[22] <estee.torok@gmail.com>

Ben Warne MB BChir MRCP[23] <ben.warne@addenbrookes.nhs.uk>

Mark Yates MBChB[13] <mark.yates@kcl.ac.uk>

Nicholas J Matheson PhD MRCP[14,23,24,25] <njm25@cam.ac.uk>

Li Su[26] PhD <li.su@mrc-bsu.cam.ac.uk >

Sofia S. Villar[26] PhD <sofia.villar@mrc-bsu.cam.ac.uk >

Grant D Stewart[b] MBChB PhD[4,27] <gds35@cam.ac.uk>

Mark Toshner*[b] MD FRCP[24,28] <mrt34@medschl.cam.ac.uk>

[a]Co-first author/contributed equally

[b]Joint senior authors/contributed equally

Affiliations

[1]Department of Clinical Neurosciences, University of Cambridge, UK

[2]Harvard Medical School, Harvard University, Boston, MA

[3]Division of Infection, University College London Hospital NHS Trust, UK

[4]Cancer Research UK Urological Malignancies Programme, Department of Oncology, University of Cambridge, UK

[5]Department of Cardiology, Hammersmith Hospital, Imperial College Healthcare NHS Trust, UK

[6]Newcastle University, Newcastle, UK

[7]Department of infection and inflammation research, Cambridge University Hospitals NHS Foundation Trust, UK

[8]Hospital for Tropical Diseases, University College London Hospital NHS Trust, UK

For peer review only

[9]MRC Clinical Trials Unit, University College London Hospital NHS Trust, UK

[10]Institute for Global Health, University College London, UK

[11]Faculty of Medicine, National Heart & Lung Institute, Imperial College London

[12]Department of Respiratory Medicine, Cambridge University Hospitals NHS Foundation Trust, Cambridge, UK

[13]Centre for Rheumatic Diseases, King's College London, London

[14]Department of Infectious Diseases, Cambridge University Hospitals NHS Foundation Trust, Cambridge, UK

[15]Research & Development, North Tees Hospital, Stockton-on-Tees, UK

[16]Imperial College London, National Heart and Lung Institute, UK

[17]Imperial College, London, UK

[18]Cambridge Urology Translational Research and Clinical Trials Department, Cambridge University Hospitals NHS Foundation Trust, UK

[19]School of Clinical Medicine, Office for Translational Research, University of Cambridge, UK

[20]Department of Respiratory Medicine, North Tees Hospital, Stockton-on-Tees, UK

[21]Division of Pathology, University College London Hospital NHS Trust, UK

[22]University of Cambridge, Cambridge, UK

[23]Cambridge Institute of Therapeutic Immunology and Infectious Disease, University of Cambridge, UK

[24]Department of Medicine, University of Cambridge, UK

[25]NHS Blood and Transplant, Cambridge, UK

[26]MRC Biostatistics Unit, University of Cambridge School of Clinical Medicine, Cambridge Institute of Public Health, UK

[27]Department of Surgery, University of Cambridge, UK

For peer review only

For peer review only - http://bmjopen.bmj.com/site/about/guidelines.xhtml

[28]NIHR Respiratory Translational Research Collaboration, UK

***Corresponding author**

Mark Toshner MD MRCP

University Lecturer

University of Cambridge School of Clinical Medicine

Department of Medicine

Box 157, Addenbrooke's Hospital

Hills Road

Cambridge CB2 0QQ

Tel: 01223 331666

Email: mrt34@medschl.cam.ac.uk

Word count: 2523

For peer review only

Structured abstract

Objectives: To analyse enrolment to interventional trials during the first wave of the coronavirus 2019 (COVID-19) pandemic in England and describe the barriers to successful recruitment in the circumstance of a further wave or future pandemics.

Design: We analysed registered interventional COVID-19 trial data and concurrently did a prospective observational study of hospitalised patients with COVID-19 who were being assessed for eligibility to one of the RECOVERY, C19-ACS or SIMPLE trials.

Setting: Interventional COVID-19 trial data were analysed from the clinicaltrials.gov and ISRCTN databases on July 12, 2020. The patient cohort was taken from 5 centres in a respiratory NIHR network. Population and modelling data were taken from published reports from the UK government and MRC biostatistics unit.

Participants: 2,082 consecutive admitted patients with laboratory-confirmed SARS-CoV-2 infection from March 27, 2020 were included.

Main outcome measures: Proportions enrolled, and reasons for exclusion from the aforementioned trials. Comparisons of trial recruitment targets with estimated feasible recruitment numbers.

Results: Analysis of trial registration data for COVID-19 treatment studies enrolling in England showed that by July 12, 2020, 29,142 participants were needed. In the observational study, 430 (20.7%) proceeded to randomisation. 82 (3.9%) declined participation, 699 (33.6%) were excluded on clinical grounds, 363 (17.4%) were medically fit for discharge, and 153 (7.3%) were receiving palliative care. With 111,037 people hospitalised with COVID-19 in England by July 12, 2020, we determine that 22,985 people were potentially suitable for trial enrolment. We estimate a UK hospitalisation rate of 2.38%, and that another 1.25 million infections would be required to meet recruitment targets of ongoing trials.

For peer review only - http://bmjopen.bmj.com/site/about/guidelines.xhtml

Conclusions: Feasible recruitment rates, study design, and proliferation of trials can limit the number, and size, that will successfully complete recruitment. We consider that fewer, more appropriately designed trials, prioritising cooperation between centres would maximise productivity in a further wave.

For peer review only

For peer review only - http://bmjopen.bmj.com/site/about/guidelines.xhtml

Strengths and limitations of this study

- We comprehensively analysed clinical trial registry data to quantify the number of participants required to successfully complete enrolment to interventional COVID-19 trials based in England in the first wave of the pandemic.
- We simultaneously performed a large, prospective, observational cohort study of 2,082 people hospitalised with COVID-19 to report recruitment rates across a range of secondary and tertiary centres and characterise reasons for trial exclusion.
- Using government data on COVID-19 hospitalisations, we consider the differences between the trials community's aspirations and delivery, and how this might inform our strategy in the event of a second wave.
- Our analysis is restricted to two registry databases and includes trials that started recruiting late in the first wave; we therefore likely underestimate the recruitment target and overestimate the number of eligible patients.
- Our analysis is limited to data based in England and, while we consider global trials, our conclusions may not be representative of, or readily translatable to, international cohorts.

For peer review only - http://bmjopen.bmj.com/site/about/guidelines.xhtml

Introduction

Unless a successful vaccination programme is deployed, the greatest need for coronavirus disease 2019 (COVID-19) remains effective treatments. This presents a substantial challenge. Ostensibly, the response from the experimental medicine community to the first wave has been robust, with more than 1,970 clinical trials planned, recruiting, or completed, at the time of writing.[1] This has enabled enrolment of patients to trials of drugs with known safety profiles – including lopinavir,[2] remdesivir,[3 4] hydroxychloroquine[5 6] and tocilizumab[7] – and led to positive results, such as the 12.1% absolute risk reduction in mortality among ventilated patients treated with dexamethasone.[8]

However, while many of these trials have been pragmatic in terms of selection criteria, the proportion of hospitalised COVID-19 patients being recruited to clinical trials is lower than might have been anticipated; the authors of the RECOVERY trial recently estimated a 10% recruitment rate in the UK.[9] Meanwhile, in areas where public health measures have limited viral transmission, trials have terminated early on account of under recruitment.[10 11] With mounting concern about an ensuing second wave of infection,[12 13] it is increasingly important to learn lessons from the first, and consider the number, size and design of clinical trials that can feasibly be completed.

We hypothesised that the proliferation of SARS-CoV-2 interventional studies during the pandemic and under recognised barriers to recruitment of COVID-19 patients led to unachievable recruitment targets in England. We used data from clinical trial registry databases to quantify recruitment targets and concurrently studied recruitment rates, including reasons for exclusion, across 5 centres enrolling patients at the peak of the first

For peer review only

For peer review only - http://bmjopen.bmj.com/site/about/guidelines.xhtml

wave of the pandemic. In conjunction with publicly available data from the UK government, we consider the differences between the trials community's aspirations and delivery, and how this might inform our strategy if there were a second wave.

For peer review only

For peer review only - http://bmjopen.bmj.com/site/about/guidelines.xhtml

Methods

Establishing recruitment targets for registered trials during first wave

COVID-19 clinical studies registered on clinicaltrials.gov or the International Standard Randomized Controlled Trial Number (ISRCTN) databases were identified and study data downloaded on July 12, 2020. Data for trials based in England, multinational trials with centres in England, and global trials were extracted in turn. Cross-registered studies were identified and accounted for once in the analysis. A manual review determined whether sponsors were academic, non-academic or mixed. Trials were excluded if they were labelled as terminated, withdrawn or suspended. Data for interventional trials examining treatment and prevention were documented, but only trials of COVID-19 treatments were used in the analysis. Analyses were performed using RStudio Version 1.2.5042.

Observational study of recruitment of hospitalised patients

We performed a prospective observational study of 2,082 consecutive patients with SARS-CoV-2 infection at 5 hospitals affiliated to the NIHR-Translational Research Collaboration with representation from secondary and tertiary centres: Cambridge University Hospitals NHS Foundation Trust (CUHFT), Cambridge; Imperial College Healthcare, University College Hospital and King's College Hospitals, London; and University Hospital of North Tees, Middlesbrough. Subjects were admitted and eligibility assessed for: RECOVERY (ISRCTN50189673), C19-ACS (NCT04333407) or SIMPLE (NCT04292730/NCT04292899). CUHFT local R&D approval was undertaken.

Demographic and clinical data were collected by contemporaneous review of potential participants' case notes. A categorical approach subdivided primary reasons subjects were

For peer review only - http://bmjopen.bmj.com/site/about/guidelines.xhtml

not enrolled into: (a) clinical grounds (screening or treating physician judgement that comorbidity or other reason for admission was more critical to patient outcome than COVID-19), (b) medically fit for discharge, (c) receiving end of life care, (d) lack of capacity, (e) patient refusal, (f) interactions with trial drugs, or (g) already on mechanical ventilation. Though already being on mechanical intervention was not an exclusion criterion for RECOVERY, patients categorised as excluded on these grounds were ineligible on account of competing, intensive care-based, studies.

Establishing feasible recruitment for registered trials during first wave

Using publicly available UK government data of the numbers of patients with COVID-19 admitted to English hospitals during the first wave between March 17 and August 5, 2020,[14] and the recruitment rate (with 95% confidence interval (CI) for one sample proportion with continuity correction) from the aforementioned observational study, we estimated a maximum bound for the accumulated feasible recruitment during that time. Simultaneously, we used the estimated cumulative number of infected cases in England by 12 July provided by MRC Biostatistics Unit at the University of Cambridge[15] to calculate an approximate hospitalisation rate in England among COVID-19 infections. We based our estimates on data from centres in England as the infection rate estimates were more reliable, hospitalisation criteria were different in Wales,[14] and the 5 hospitals included in this study are all from England.

Patient and public involvement

This was a time-critical study in response to a Public Health Emergency of International Concern. Patients or the public were not involved in the design, conduct, or reporting of this research.

For peer review only - http://bmjopen.bmj.com/site/about/guidelines.xhtml

For peer review only

For peer review only - http://bmjopen.bmj.com/site/about/guidelines.xhtml

Results

Establishing recruitment targets to registered trials during first wave

Clinical trial registry data were downloaded on July 12, 2020; 28 interventional studies were included in our analysis of those registered in England. 22 (78%) were academically sponsored, 5 (18%) were non-academically sponsored and 1 (4%) was mixed. The first registration date of a COVID-19 treatment trial in England was March 22; the earliest registered start date was March 12. Analysis of recruitment targets for each trial revealed that 46,154 participants would be required to complete recruitment to all studies in England (Table 1): 17,012 people are required for trials of prophylactic drugs to prevent COVID-19, while 29,142 are needed for those treating established COVID-19 (Table 1). The median (IQR) treatment trial recruitment target was 195 (50-793).

By contrast, the global situation is such that 1,107 registered interventional trials were ongoing or completed, requiring 566,872 patients to be randomised to allow their completion; 306,426 of these are needed for trials of COVID-19 treatments (Figure 1A and 1B). These trials are geographically clustered in China, North America and Europe (Figure 1C).

Observational study of clinical trial enrolment

From March 27 to May 22, 2020 a total of 2,082 consecutive patients were included across the 5 sites (Table 2). Age and sex data were available for 1,971 patients: the median (IQR) age was 71 (58-82) and 56.2% were male. Across the four trials, 430 (20.7%, 95% CI [18.95%, 22.47%]) proceeded to randomisation.

Of the remaining 1,652 patients, 82 (3.9%) declined participation, 363 (17.4%) were medically fit for discharge, 153 (7.3%) were receiving end of life care and 106 (5.1%) were

For peer review only - http://bmjopen.bmj.com/site/about/guidelines.xhtml

mechanically ventilated at the time of screening. In 699 (33.6%) patients, the screening or treating physician determined that the potential participant should not be enrolled on account of clinical grounds or trial exclusion criteria.

Establishing feasible recruitment for registered trials during first wave

By combining these observed recruitment rates with publicly reported hospitalisation data (between March 17, and July 12, 2020), we estimated a maximum upper bound for the accumulated feasible recruitment for registered trials of COVID-19 treatments in England during the first wave (Figure 2).

The estimated number of cumulative infected cases by 12 July reported by MRC Biostatistics Unit is 4.67 million with a 95% credible interval [3.76, 6.04]. Combined with the number of cumulative admitted patients in England by 12 July from government data (i.e. 111,037 hospital admissions), this gives an approximate hospitalisation rate 2.38% [1.84%, 2.95%] in England during the first wave.

Our analysis indicates that by July 12th, 6,158 patients might still be needed to meet the total recruitment targets for currently recruiting clinical trials. If considering uncertainty in recruitment rate estimate reflected by 95% CI [18.95%, 22.47%], 4,192-8,100 patients might be required to meet recruitment target. Assuming the recruitment rate 20.7%, this implies that 29,749 hospitalised patients would need to be screened for these trials to complete recruitment. With the approximate hospitalisation rate 2.38% in England as observed in the first wave, this would require 1.249 million patients to be infected.

For peer review only - http://bmjopen.bmj.com/site/about/guidelines.xhtml

With the daily infection rate for UK estimated to be 3,310 (95% credible interval [2440, 4460]) on 12 July,[15] it is highly unlikely such a large number of hospitalisations would occur unless there is an increase in the infection numbers (or a second wave). Indeed, incorporating hospitalisation data to August 5, 2020, shows minimal progress toward the recruitment target, assuming no new trials were approved after July 12, 2020 (Figure 2B).

For peer review only

For peer review only - http://bmjopen.bmj.com/site/about/guidelines.xhtml

Discussion

We found that the proliferation of clinical trials[1] in response to the first wave of the COVID-19 pandemic in England required 29,142 participants to complete enrolment to those registered with a trials database. Globally, 306,426 participants are required to meet recruitment targets for trials of treatments of COVID-19. Meanwhile, in our multicentre prospective observational cohort study of patients admitted to hospital with laboratory-confirmed COVID-19, 79.3% of potential participants were not recruited to a clinical trial; the reasons for excluding patients were varied and clarify the challenges faced in both general hospitals and well-resourced centres experienced in experimental medicine. Our experience is consistent with the general literature on clinical trial recruitment where many factors have been posited to contribute to heterogeneity of recruitment.[16] With 111,037 people hospitalised in England between March 17 and July 12, 2020, our net recruitment rate suggests that 22,985 (21,042-24,950 if taking into account uncertainty in recruitment rate estimate by random errors) would have been potentially suitable for selection in the first wave. However, this is clearly an overestimate, given that it would require each of these individuals to be hospitalised in geographical locations where medical centres were undertaking these trials. In the first wave, most general clinical trials infrastructure was mothballed for normal activity and therefore easily seconded towards COVID-19 and this may not be the case in subsequent "waves". It must also be recalled that most recruitment in the first wave was undertaken as hospitals were actively reconfiguring services. A stable hospital infrastructure may positively impact on ease of delivery in the future. Nevertheless, unless there is a second wave it is highly unlikely that the total recruitment target will be met in any reasonable timeframe.

For peer review only - http://bmjopen.bmj.com/site/about/guidelines.xhtml

Strengths of our study are that our analyses of registry and population databases utilised the largest and most robust data available. Meanwhile, our observational study applied a large cohort size, prospective data acquisition, and recorded detailed reasons for excluding patients. By using both secondary and tertiary care centres, we believe our results are generalisable to other hospitals in the UK. Also, by following studies with minimal selection criteria, particularly in the RECOVERY trial, we reduced the chance of underestimating trial recruitment. Our study does have limitations. First, our predictions were based on registry data for studies based in England alone; we did not include the numbers of participants required to be recruited into multinational trials in which the English centres were involved. The result is that we have likely underestimated the trial recruitment target for England and, by extension, the gap between this and the number of participants available. Second, although we used hospitalisation data from 17 March 2020, as this was the time the UK government commenced public reporting of COVID-19 admissions, all trials included in our registry analysis were not recruiting at that stage; the earliest start date for a trial registered in England was March 12, 2020, but the last trial start date was not until July 7, 2020. In this sense, using cumulative number of admitted patients in our prediction is optimistic. Third, we only included the two registry datasets in most widespread use, and so may have further underestimated the number of studies and participants required. Fourth, the 95% CI for recruitment rate estimate only reflects the uncertainty due to random errors in the data, it does not consider the uncertainty due to unrepresentativeness of data from the 5 hospital centres in our study. Finally, although we illustrate the scale of trial recruitment required globally, the populations tested may not be representative of, or translatable to, international cohorts.

Our study is the first to characterise the suitability and barriers for trial enrolment for a complete cohort of hospitalised patients with COVID-19. Results of trials published to date

For peer review only - http://bmjopen.bmj.com/site/about/guidelines.xhtml

convey a different message: interventional studies of lopinavir and remdesivir, for example, have recruitment rates ranging from 55.7%-96.0%.[2-4] This difference is most likely explained by the different denominators used in our calculations: the consort diagrams in clinical trials are unlikely to include every single patient hospitalised with a positive test. Instead, our results align with or exceed other centres, such as the 10% recruitment rate to RECOVERY.[9] During the 2013-16 Ebola Virus Disease (EVD) epidemic in west Africa, most clinical trials during that crisis either started too late to enrol sufficient case numbers or were simply unable to reach their recruitment targets.[17] Our study shows that trials in England started recruiting relatively quickly, however many are highly unlikely to recruit on time; we conclude that starting early is important but not enough to ensure recruitment targets are met. Finally, it is notable that our calculated hospitalisation rate of 2.38% is lower than that observed in Wuhan,[18] which if applied to the UK age structure,[19] is equivalent to approximately 5.8%.

The disparity between the realistic recruitment rates and high requirements we report leads us to conclude that the scientific community should be increasingly selective in the number, size and design of clinical trials deployed in the COVID-19 pandemic; our findings have meaning for those planning single trials, and those strategizing the national response. Potential solutions include practical changes to trial design, for instance capturing patients earlier in their disease path, and adopting dynamic and adaptive trial designs.[20] Yet, such measures are unlikely to bridge the currently estimated large recruitment gap. Instead, it may be necessary for healthcare authorities and policy makers to foster more academic cooperation to prioritise compounds, prevent duplication and, perhaps more radically, perform real-time meta-analyses of ongoing trials of the same therapies and provide stop/go recommendations across trials to rationalise treatment and prevent multiple studies delaying reporting.[21] Indeed, proposals have been forthcoming for mechanisms by which data from different trials might

For peer review only - http://bmjopen.bmj.com/site/about/guidelines.xhtml

be shared and analysed in a robust and scientifically meaningful way.[22] These conclusions are not dissimilar to reflections from the Ebola pandemic, when there was a strong call for strengthening and coordinating research efforts in response to outbreaks of emerging infectious diseases.[23 24] For planning future trials and deriving realistic recruitment targets, real-time tracking of the pandemic, as data accumulate over time, is essential to plan research in response of an emerging epidemics outbreak. The Medical Research Council (MRC) Biostatistics Unit regularly nowcast and forecast COVID-19 infections and deaths.[15] This information feeds directly to SAGE sub-group, Scientific Pandemic Influenza sub-group on Modelling (SPI-M) and to regional PHE teams. This same data could be used to establish realistic recruitment trends to inform, monitor and coordinate research efforts both for treatment and prevention trials.

Multiple questions remain for future research. In particular, it remains unclear how relaxing of non-pharmacological interventions will affect transmission rates, and therefore the achievability of remaining recruitment to these trials. It is also unknown how a second wave would evolve, and whether more or fewer people will develop the illness than was seen in the first. Nonetheless, we conclude that clinical trialists and healthcare authorities must consider the recruitment challenges when determining the feasibility of clinical trials in a second wave and urgently rationalise those currently active.

For peer review only - http://bmjopen.bmj.com/site/about/guidelines.xhtml

Acknowledgements

We acknowledge the use of original data from the UK government (https://coronavirus.data.gov.uk/) and infection case estimates of England in the nowcasting and forecasting report provided by the MRC Biostatistics Unit.

Footnotes

Contributors: Dr Cunniffe and Dr Toshner accept full responsibility for the work and conduct of the study, had access to the data, and controlled the decision to publish. Dr Cunniffe and Simon Gunter contributed equally as co-first authors. Dr Toshner and Prof Stewart contributed equally as joint senior authors.

Study concept and design: Cunniffe, Gunter, Stewart, Su, Villar, Toshner

Acquisition, analysis or interpretation of data: Cunniffe, Gunter, Stewart, Su, Villar, Toshner, Brown, Burge, Coyle, De-Soyza, Dymond, Esmail, Francis, Jacqui Galloway, James Galloway, Gkrania-Klotsas, Greenaway, Katritsis, Kanagaratnam, Knolle, Leonard, McIntyre, Prudon, Rampling, Torok, Warne, Yates and Matheson

Drafting of manuscript: Cunniffe, Gunter, Stewart, Su, Villar, Toshner

Critical revision of the manuscript: Cunniffe, Gunter, Stewart, Su, Villar, Toshner, Brown, Burge, Coyle, De-Soyza, Dymond, Esmail, Francis, Jacqui Galloway, James Galloway PhD, Gkrania-Klotsas, Greenaway, Katritsis, Kanagaratnam, Knolle, Leonard, McIntyre, Prudon, Rampling, Torok, Warne and Matheson

Statistical analysis: Cunniffe, Gunter, Warne, Matheson

Competing interests: None declared.

For peer review only - http://bmjopen.bmj.com/site/about/guidelines.xhtml

For peer review only

Transparency statement: Dr Toshner, the manuscript's guarantor, affirms that the manuscript is an honest, accurate, and transparent account of the study being reported; that no important aspects of the study have been omitted; and that any discrepancies from the study as originally planned (and, if relevant, registered) have been explained.

Funding: Dr Burge and Prof Stewart declare funding from a Cancer Research UK Major Centre award (C9685/A25117). Dr Coyle declares funding from the British Heart Foundation. Dr Gkrania-Klotsas is supported by an NIHR Greenshoots personal Award from the Clinical Research Network. Prof Kanagaratnam declares funding from the Imperial College COVID-19 Research Fund. Dr Knolle declares funding from MRC CARP. Dr Torok declares support from a Clinician Scientist Fellowship (Academy of Medical and the Health Foundation) and the NIHR Cambridge Biomedical Research Centre. Dr Warne receives funding from the National Institute for Health Research Cambridge Biomedical Research Centre at the Cambridge University Hospitals NHS Foundation Trust. Dr Yates' salary is funded by The British Society for Rheumatology and Versus Arthritis. Dr Matheson declares funding from MRC (CSF MR/P008801/1) and NHSBT (WPA15-02). Dr Li declares funding from the Medical Research Council programme MRC_MC_UU_00002/10. Dr Villar declares funding from the Medical Research Council (grant number: MC_UU_00002/15). Dr Toshner is funded by the NIHR Cambridge Biomedical Research Centre. This work was enabled by co-operation of the NIHR Respiratory Translational Research Collaboration.

Ethical approval: Data were acquired within the ethical approvals of the aforementioned trials.

For peer review only - http://bmjopen.bmj.com/site/about/guidelines.xhtml

Data sharing: All relevant data is included in the manuscript. Data from the clinical trials registries, UK government, and MRC biostatistics unit are available on public websites.

For peer review only

For peer review only - http://bmjopen.bmj.com/site/about/guidelines.xhtml

Figure legends

Figure 1. The proliferation of global clinical trials in response to COVID-19. A: cumulative number of enrolling studies registered with clinicaltrials.gov or ISRCTN until Jul 12, 2020, subdivided by those testing drugs for COVID-19 treatment and prevention. B*:* cumulative number of participants required to meet recruitment targets for registered clinical trials. C: geographical distribution of COVID-19 clinical trials.

Figure 2. Feasibility of achieving target recruitment in England for COVID-19 interventional studies. A: cumulative number of enrolling studies in England registered with clinicaltrials.gov or ISRCTN until July 12, 2020, subdivided by those testing drugs for COVID-19 treatment and prevention. B*:* cumulative number of participants required to meet recruitment targets for registered COVID-19 treatment trials until July 12, 2020, and predicted number of patients whom would have been eligible for randomisation (grey shaded area represents point-wise 95% confidence band for the predictive cumulative number of eligible patients using the lower and upper value of 95% confidence interval for the recruitment rate estimate with continuity correction). The reduction in the infection rate in England means that the recruitment target at July 12 is unlikely to be reached unless there is a second wave; further illustrated by extending hospitalisation data to August 5, 2020.

For peer review only

For peer review only - http://bmjopen.bmj.com/site/about/guidelines.xhtml

Tables

	Number of Trials	Number of Participants
**Global Trials**		
Prevention	172	260,446
Treatment	935	306,426
Total	1,107	566,872
**UK Multi-National and National Trials**		
Prevention	11	97,272
Treatment	38	44,362
Total	49	141,634
**England Trials**		
Prevention	8	17,012
Treatment	20	29,142
Total	28	46,154

Table 1: Summary of number of trials and required numbers of participants

For peer review only - http://bmjopen.bmj.com/site/about/guidelines.xhtml

	RECOVERY				COMBINATION*	C19-ACS	SIMPLE	**Total**
**Total screened per centre**	281	83	415	Total (779)	445	784	74	2,082
**Number recruited (%)**	35 (12.5)	16 (19.3)	185 (44.6)	236 (30.3)	124 (27.9)	56 (7.1)	14 (18.9)	**430 (20.7)**
Refused participation (%)	10 (3.6)	19 (22.9)	16 (3.9)	45 (5.8)	8 (1.8)	29 (3.7)	0 (0.0)	82 (3.9)
Clinical grounds/trial exclusion criteria(%)	83 (29.5)	15 (18.1)	40 (9.6)	138 (17.7)	167 (37.5)	365 (46.6)	29 (39.2)	699 (33.6)
Lacked capacity (%)	22 (7.8)	0 (0.0)	1 (0.2)	23 (3.0)	16 (3.6)	98 (12.5)	0 (0.0)	137 (6.6)
Mechanical ventilation (%)	37 (13.2)	7 (8.4)	0 (0.0)	44 (5.6)	7 (1.6)	48 (6.1)	7 (9.5)	106 (5.1)
Drug interactions (%)	12 (4.3)	2 (2.4)	0 (0.0)	14 (1.8)	2 (0.4)	1 (0.1)	0 (0.0)	17 (0.8)
Medically fit for discharge (%)	55 (19.6)	14 (16.9)	77 (18.6)	146 (18.7)	65 (14.6)	136 (17.3)	16 (21.6)	363 (17.4)
Palliative care (%)	19 (6.8)	7 (8.4)	61 (14.7)	87 (11.2)	8 (1.8)	51 (6.5)	7 (9.5)	153 (7.3)
Not approached or considered (%)	8 (2.8)	3 (3.6)	35 (8.4)	46 (5.9)	48 (10.8)	0 (0.0)	1 (1.4)	95 (4.6)
**Total not recruited (%)**	246 (87.5)	67 (80.7)	230 (55.4)	543 (69.7)	321 (72.1)	728 (92.9)	60 (81.1)	**1,652 (79.3)**

Table 2: screening data for 2,082 consecutive patients with laboratory-confirmed SARS-CoV-2 admitted to one of 5 centres. *centre screened concurrently to both RECOVERY and SIMPLE: moderate and severe trials.

For peer review only - http://bmjopen.bmj.com/site/about/guidelines.xhtml

References

1. Thorlund K, Dron L, Park J, et al. A real-time dashboard of clinical trials for COVID-19. *The Lancet Digital Health* 2020;2(6):e286-e87. doi: 10.1016/S2589-7500(20)30086-8

2. Cao B, Wang Y, Wen D, et al. A Trial of Lopinavir–Ritonavir in Adults Hospitalized with Severe Covid-19. *New England Journal of Medicine* 2020;382(19):1787-99. doi: 10.1056/NEJMoa2001282

3. Beigel JH, Tomashek KM, Dodd LE, et al. Remdesivir for the Treatment of Covid-19 — Preliminary Report. *New England Journal of Medicine* 2020 doi: 10.1056/NEJMoa2007764

4. Wang Y, Zhang D, Du G, et al. Remdesivir in adults with severe COVID-19: a randomised, double-blind, placebo-controlled, multicentre trial. *The Lancet* 2020;395(10236):1569-78. doi: 10.1016/S0140-6736(20)31022-9

5. Geleris J, Sun Y, Platt J, et al. Observational Study of Hydroxychloroquine in Hospitalized Patients with Covid-19. *New England Journal of Medicine* 2020;382(25):2411-18. doi: 10.1056/NEJMoa2012410

6. Gautret P, Lagier J-C, Parola P, et al. Hydroxychloroquine and azithromycin as a treatment of COVID-19: results of an open-label non-randomized clinical trial. *International Journal of Antimicrobial Agents* 2020:105949. doi: https://doi.org/10.1016/j.ijantimicag.2020.105949

7. Zhang C, Wu Z, Li J-W, et al. Cytokine release syndrome in severe COVID-19: interleukin-6 receptor antagonist tocilizumab may be the key to reduce mortality. *International Journal of Antimicrobial Agents* 2020;55(5):105954. doi: https://doi.org/10.1016/j.ijantimicag.2020.105954

For peer review only

For peer review only - http://bmjopen.bmj.com/site/about/guidelines.xhtml

8. The RECOVERY Collaborative Group. Dexamethasone in Hospitalized Patients with Covid-19 — Preliminary Report. *New England Journal of Medicine* 2020 doi: 10.1056/NEJMoa2021436

9. Wilkinson E. RECOVERY trial: the UK covid-19 study resetting expectations for clinical trials. *BMJ* 2020;369:m1626. doi: 10.1136/bmj.m1626

10. Norrie JD. Remdesivir for COVID-19: challenges of underpowered studies. *The Lancet* 2020;395(10236):1525-27. doi: 10.1016/S0140-6736(20)31023-0

11. Li L, Zhang W, Hu Y, et al. Effect of Convalescent Plasma Therapy on Time to Clinical Improvement in Patients With Severe and Life-threatening COVID-19: A Randomized Clinical Trial. *JAMA* 2020;324(5):460-70. doi: 10.1001/jama.2020.10044

12. Xu S, Li Y. Beware of the second wave of COVID-19. *The Lancet* 2020;395(10233):1321-22. doi: 10.1016/S0140-6736(20)30845-X

13. Petersen E, Koopmans M, Go U, et al. Comparing SARS-CoV-2 with SARS-CoV and influenza pandemics. *The Lancet Infectious Diseases* doi: 10.1016/S1473-3099(20)30484-9

14. Publich Health England. Coronavirus (COVID-19) in the UK 2020 [Available from: https://coronavirus.data.gov.uk/healthcare] accessed August 5 2020.

15. MRC Biostatistics Unit. COVID-19: nowcast and forecast 2020 [Available from: https://www.mrc-bsu.cam.ac.uk/now-casting/report-on-nowcasting-and-forecasting-6th-august-2020/] accessed August 14 2020.

16. Thoma A, Farrokhyar F, McKnight L, et al. Practical tips for surgical research: how to optimize patient recruitment. *Can J Surg* 2010;53(3):205-10. [published Online First: 2010/05/29]

For peer review only - http://bmjopen.bmj.com/site/about/guidelines.xhtml

17. Rojek A, Horby P, Dunning J. Insights from clinical research completed during the west Africa Ebola virus disease epidemic. *The Lancet Infectious Diseases* 2017;17(9):e280-e92. doi: 10.1016/S1473-3099(17)30234-7

18. Verity R, Okell LC, Dorigatti I, et al. Estimates of the severity of coronavirus disease 2019: a model-based analysis. *The Lancet Infectious Diseases* 2020;20(6):669-77. doi: 10.1016/S1473-3099(20)30243-7

19. Office of national statistics. Old age structure variant - UK population in age groups 2018 [Available from: https://www.ons.gov.uk/peoplepopulationandcommunity/populationandmigration/populationprojections/datasets/tablel21oldagestructurevariantukpopulationinagegroups] accessed May, 28 2020.

20. Brueckner M, Titman A, Jaki T, et al. Performance of different clinical trial designs to evaluate treatments during an epidemic. *PLOS ONE* 2018;13(9):e0203387. doi: 10.1371/journal.pone.0203387

21. Bauchner H, Fontanarosa PB. Randomized Clinical Trials and COVID-19: Managing Expectations. *JAMA* 2020;323(22):2262-63. doi: 10.1001/jama.2020.8115

22. Petkova E, Antman EM, Troxel AB. Pooling Data From Individual Clinical Trials in the COVID-19 Era. *JAMA* 2020 doi: 10.1001/jama.2020.13042

23. World Health Organisation. Report of the Ebola Interim Assessment Panel - May 2015, 2015.

24. National Academies of Sciences E, Medicine. Integrating Clinical Research into Epidemic Response: The Ebola Experience. Washington, DC: The National Academies Press 2017.

For peer review only

For peer review only - http://bmjopen.bmj.com/site/about/guidelines.xhtml

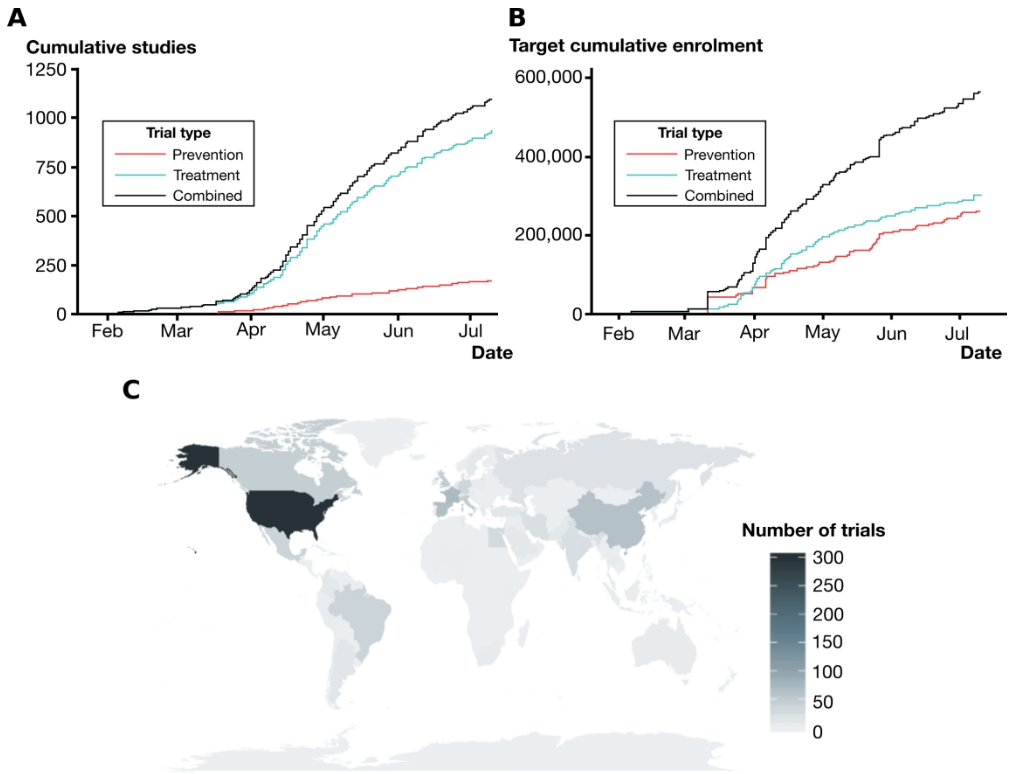

Figure 1. The proliferation of global clinical trials in response to COVID-19. A: cumulative number of enrolling studies registered with clinicaltrials.gov or ISRCTN until Jul 12, 2020, subdivided by those testing drugs for COVID-19 treatment and prevention. B: cumulative number of participants required to meet recruitment targets for registered clinical trials. C: geographical distribution of COVID-19 clinical trials.

193x153mm (300 x 300 DPI)

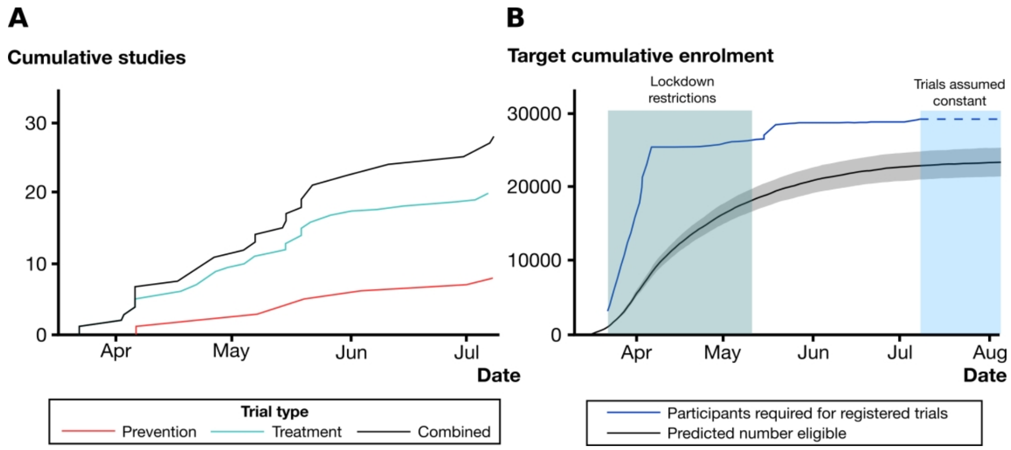

Figure 2. Feasibility of achieving target recruitment in England for COVID-19 interventional studies. A: cumulative number of enrolling studies in England registered with clinicaltrials.gov or ISRCTN until July 12, 2020, subdivided by those testing drugs for COVID-19 treatment and prevention. B: cumulative number of participants required to meet recruitment targets for registered COVID-19 treatment trials until July 12, 2020, and predicted number of patients whom would have been eligible for randomisation (grey shaded area represents point-wise 95% confidence band for the predictive cumulative number of eligible patients using the lower and upper value of 95% confidence interval for the recruitment rate estimate with continuity correction). The reduction in the infection rate in England means that the recruitment target at July 12 is unlikely to be reached unless there is a second wave; further illustrated by extending hospitalisation data to August 5, 2020.

184x80mm (300 x 300 DPI)

STROBE Statement—Checklist of items that should be included in reports of ***cohort studies***

	Item No	Recommendation	Page No
**Title and abstract**	1	(*a*) Indicate the study's design with a commonly used term in the title or the abstract<br>(*b*) Provide in the abstract an informative and balanced summary of what was done and what was found	1,5
**Introduction**			
Background/rationale	2	Explain the scientific background and rationale for the investigation being reported	8,9
Objectives	3	State specific objectives, including any prespecified hypotheses	5,8,9
**Methods**			
Study design	4	Present key elements of study design early in the paper	5,8,9
Setting	5	Describe the setting, locations, and relevant dates, including periods of recruitment, exposure, follow-up, and data collection	5,10,11
Participants	6	(*a*) Give the eligibility criteria, and the sources and methods of selection of participants. Describe methods of follow-up<br>(*b*) For matched studies, give matching criteria and number of exposed and unexposed	5,10,11
Variables	7	Clearly define all outcomes, exposures, predictors, potential confounders, and effect modifiers. Give diagnostic criteria, if applicable	5,10,11
Data sources/ measurement	8*	For each variable of interest, give sources of data and details of methods of assessment (measurement). Describe comparability of assessment methods if there is more than one group	5,10,11
Bias	9	Describe any efforts to address potential sources of bias	10,11
Study size	10	Explain how the study size was arrived at	10,11
Quantitative variables	11	Explain how quantitative variables were handled in the analyses. If applicable, describe which groupings were chosen and why	10,11
Statistical methods	12	(*a*) Describe all statistical methods, including those used to control for confounding<br>(*b*) Describe any methods used to examine subgroups and interactions<br>(*c*) Explain how missing data were addressed<br>(*d*) If applicable, explain how loss to follow-up was addressed<br>(*e*) Describe any sensitivity analyses	10,11
**Results**			
Participants	13*	(a) Report numbers of individuals at each stage of study—eg numbers potentially eligible, examined for eligibility, confirmed eligible, included in the study, completing follow-up, and analysed<br>(b) Give reasons for non-participation at each stage<br>(c) Consider use of a flow diagram	5,13
Descriptive data	14*	(a) Give characteristics of study participants (eg demographic, clinical, social) and information on exposures and potential confounders<br>(b) Indicate number of participants with missing data for each variable of interest<br>(c) Summarise follow-up time (eg, average and total amount)	5,13
Outcome data	15*	Report numbers of outcome events or summary measures over time	5,13,14,15

For peer review only

For peer review only - http://bmjopen.bmj.com/site/about/guidelines.xhtml

Main results	16	(*a*) Give unadjusted estimates and, if applicable, confounder-adjusted estimates and their precision (eg, 95% confidence interval). Make clear which confounders were adjusted for and why they were included<br>(*b*) Report category boundaries when continuous variables were categorized<br>(*c*) If relevant, consider translating estimates of relative risk into absolute risk for a meaningful time period	13-15
Other analyses	17	Report other analyses done—eg analyses of subgroups and interactions, and sensitivity analyses	14-15
**Discussion**			
Key results	18	Summarise key results with reference to study objectives	16
Limitations	19	Discuss limitations of the study, taking into account sources of potential bias or imprecision. Discuss both direction and magnitude of any potential bias	18
Interpretation	20	Give a cautious overall interpretation of results considering objectives, limitations, multiplicity of analyses, results from similar studies, and other relevant evidence	17,18,19
Generalisability	21	Discuss the generalisability (external validity) of the study results	16,17,18
**Other information**			
Funding	22	Give the source of funding and the role of the funders for the present study and, if applicable, for the original study on which the present article is based	22

*Give information separately for exposed and unexposed groups.

Note: An Explanation and Elaboration article discusses each checklist item and gives methodological background and published examples of transparent reporting. The STROBE checklist is best used in conjunction with this article (freely available on the Web sites of PLoS Medicine at http://www.plosmedicine.org/, Annals of Internal Medicine at http://www.annals.org/, and Epidemiology at http://www.epidem.com/). Information on the STROBE Initiative is available at http://www.strobe-statement.org.

For peer review only
