# Supplementary material for: How achievable are COVID-19 clinical trial recruitment targets? A UK observational cohort study and trials registry analysis
Source: BMJ Open. 2020 Oct 5;10(10):e044566. doi: 10.1136/bmjopen-2020-044566 (PMC7536634; doi:10.1136/bmjopen-2020-044566)
Supplement: Reviewer comments [file bmjopen-2020-044566.reviewer_comments.pdf]

PEER REVIEW HISTORY

BMJ Open publishes all reviews undertaken for accepted manuscripts. Reviewers are asked to complete a checklist review form (**http://bmjopen.bmj.com/site/about/resources/checklist.pdf**) and are provided with free text boxes to elaborate on their assessment. These free text comments are reproduced below.

ARTICLE DETAILS

**TITLE (PROVISIONAL)**	How achievable are COVID-19 clinical trial recruitment targets? A UK observational cohort study and trials registry analysis
**AUTHORS**	Cunniffe, Nick; Gunter, Simon; Brown, Michael; Burge, Sarah; Coyle, Clare; De Soyza, Anthony; Dymond, Tom; Esmail, Hanif; Francis, Darrel; Galloway, Jacqui; Galloway, James B; Gkrania-Klotsas, Effrossyni; Greenaway, Jane; Katritsis, George; Kanagaratnam, Prapa; Knolle, Martin; Leonard, Kelly; McIntyre, Zoe; Prudon, Ben; Rampling, Tommy; Torok, Mili; Warne, Ben; Yates, Mark; Matheson, Nicholas; Su, Li; Villar, Sofia; Stewart, Grant; Toshner, Mark

This article was not externally reviewed at BMJ Open. Protocols that have been independently assessed prior to submission to BMJ Open are usually fast-tracked to publication on the grounds that further substantial changes will not be possible. This independent assessment will usually be external, independent review for both a substantial grant award from a non-commercial or government funder and ethics approval.
